# The interplay between social environment and opportunities for physical activity within the built environment: a scoping review

**DOI:** 10.1186/s12889-024-19733-x

**Published:** 2024-08-30

**Authors:** Jens Høyer-Kruse, Eva Berthelsen Schmidt, Anne Faber Hansen, Marlene Rosager Lund Pedersen

**Affiliations:** 1 Department of Sports Science and Clinical Biomechanics, University of Southern Denmark, Odense M, 5230 Denmark; 2 Department of Teaching and Dissemination, University Library of Southern Denmark, Odense M, 5230 Denmark

**Keywords:** Social environment, Built environment, Physical activity, Health, Scoping review

## Abstract

**Background:**

The association between social and built environments plays a crucial role in influencing physical activity levels. However, a thorough understanding of their combined impact remains unclear. This scoping review seeks to clarify the interplay between social environments and opportunities for physical activity within different built environments, with a particular focus on the implications of socioeconomic status and urban planning on physical activity participation.

**Methods:**

We conducted a systematic literature search across several databases to identify studies exploring the associations between social factors, built environment characteristics, and physical activity levels. The inclusion criteria were studies published in English between 2000 and 2022, encompassing urban, suburban, and rural contexts. Thematic analysis was employed to categorise studies based on the specific aspects of the built environment they investigated (walking infrastructure, cycling infrastructure, parks and open spaces, and sports facilities) and the social determinants they examined.

**Results:**

A total of 72 studies were included in the review, illustrating a multifaceted relationship between access to physical activity opportunities and social determinants such as socioeconomic status, community engagement, and urban design. The findings highlight the significant role of socioeconomic factors and the quality of PA infrastructure in promoting or hindering PA across communities. Effective urban planning was identified as crucial in providing expanded physical activity opportunities, notably through more pedestrian-friendly environments, comprehensive cycling infrastructure, and accessible green spaces and sports facilities.

**Conclusions:**

This review emphasises the significant impact of socioeconomic status and urban planning on access to physical activity opportunities. This underscores the necessity for urban planning policies to adopt an inclusive approach, considering the varied needs of different population groups to ensure equitable access to physical activity resources. Such strategies are crucial for public health initiatives aimed at enhancing physical activity levels across diverse community sectors, offering a potential avenue to alleviate health disparities associated with inactivity.

**Supplementary Information:**

The online version contains supplementary material available at 10.1186/s12889-024-19733-x.

## Background

It is well known that physical activity (PA) is crucial to reducing chronic diseases and enhancing population health. Despite increased attention in recent years, the World Health Organization has revealed [1] that only one in four adults meets the recommendations for PA. The undeniable link between regular physical activity and overall well-being underscores the need for a comprehensive understanding of the factors influencing individuals’ engagement in PA. Two key determinants – the social environment and built environment – stand out as two of the most important factors in this intricate equation and have received increased attention in recent years [1, 2].

The built environment is essential for PA opportunities, encompassing aspects of urban and architectural design, traffic density and speed, distance to and design of venues for PA, and crime and safety [3, 4]. For example, previous studies have explored the impact of the built environment on PA, revealing how factors such as sports facilities, accessible parks, pedestrian-friendly paths, and community infrastructure can foster or hinder active lifestyles [5–8]. Similarly, social environment can be related to the level of PA, and the relationship between these two factors has been investigated in numerous studies [9–11]. Determinants of the social environment related to health include individual factors (age, gender, education, etc.), poverty and deprivation, social networks, political environment (e.g. policy), and background conditions, such as culture and economy [12]. In addition, the social environment can also be related to the landscape of the investigation, where several studies include the socioeconomic situation within the local area or neighbourhood, e.g. [13, 14].

A recent study by Wang et al. [15] conducted a scoping review and examined the interaction between built and social environments and its impact on PA. They found that built and social environments influence PA and that consideration of people’s perceptions of their surroundings can provide further insight. This approach, focusing on broader determinants of health behaviour, is consistent with the socio-ecological perspective of health behaviour, where multiple factors interact with or influence PA, including aspects of work, physical and social environments, community conditions, and policies [16]. Combining these factors in the same study has become common practice, e.g. [2, 15, 17, 18].

However, research on the interplay between social environment and *opportunities* for PA within the built environment is limited. In this regard, Brug et al. [19], stated in their study that ‘what we really need are not studies that highlight the importance of individual factors, social factors or built environmental factors in shaping nutrition and PA behaviours. We need more studies that integrate potential determinants at the environmental level and the individual levels.’ Drawing from this, our purpose is twofold: (1) to review the current literature to shed light on how social environment intersects with *opportunities* for PA within the four types of built environments inspired by McCormack and Shiell [20]: walking infrastructure, cycling infrastructure, neighbourhood parks, open spaces, and sports facilities; and (2) how the social environment combined with *opportunities* for PA within the built environment impacts PA levels. This scoping review differs from previous studies, e.g. [2, 15, 17], because despite its focus on PA levels, it also seeks to understand the differences in *opportunities* for PA within the four built environments and how they are influenced by social environment factors at the area and individual levels. Therefore, the included studies do not necessarily include measures of PA levels, but must, as a minimum, include measures to investigate the association between the built environment for PA and the social environment. Based on the significance drawn from this study, it can inform public health officials and planners.

## Methods

### Terminology of the main terms (physical activity, built environment and social environment)

Physical activity can be defined as ‘any bodily movement produced by skeletal muscle that results in energy expenditure’ [21]. In this framework, physical activity includes sports participation, active outdoor living, active transportation, recreational sports, and physical activity at work and during housework.

In this study, the built environment is defined as ‘the physical makeup of where we live, learn, work, and play – our homes, schools, businesses, streets and sidewalks, open spaces, and transportation options. The built environment may influence overall community health and individual behaviours such as physical activity and healthy eating’ [4]. This definition encompasses elements most pertinent to behaviours related to physical activity, including community design, public transport, built environment for active transportation (walking and biking), pedestrian safety, and other types of built environments in the local area, such as green areas, parks, open spaces, aesthetics and pleasantness, recreational facilities, and sports facilities [22]. Inspired by the categorisation of McCormack and Shiell [20], we divided the results of this study into four types of built environment: walking infrastructure cycling infrastructure, neighbourhood parks, open spaces, and sports facilities.

When examining the social environment, we were inspired by the social determinants of health described in the social ecological model of Gubbels et al. [16] and the definition of Blazer et al. [12] – meaning that besides our primary focus on factors of socioeconomic status (SES), including education, occupation, and income levels, this scoping review also encompasses a variety of individual factors (including age, gender, disabilities and ethnic background), social networks (including family and community), combined with policies, and the socioeconomic and cultural landscape of society with which individuals interact. For example, some of the included studies compared high-SES areas with low-SES areas, and some compared individuals with different ethnic backgrounds or income levels; that is, the social environment refers to the relationships, culture, and society with which individuals interact. Additionally, area density was used as a marker of the social environment.

In continuation of the description of social environment, in the results section, we further separate our results into individual-specific results and area-specific results focusing on either the individual factors (e.g. personal income, gender, age) of the social environment or societal or area factors (policies, density, socioeconomic, or cultural landscape of society, including area level income) of the social environment.

### Identification of studies

#### Search for core concepts related to scope and databases

To conduct this scoping review, we use the guidance for conducting systematic scoping reviews described by Peters et al. [23]. Initial scoping searches of the central issues of the research question (physical activity, social environment, and built environment) were conducted using several databases. The four databases with the most relevant preliminary search results were selected for the final search. The final literature search was performed using Global Health, Scopus, Sociological Abstracts, and SPORTdiscus. Studies published until 1 November 2019, included full English, German, Danish, Swedish, or Norwegian texts. There were no restrictions on publication year. The literature search was updated by 29th of August 2022 and pooled with the existing search results in Covidence (© 2022 Covidence). Duplicates were removed before review.

### Search strategy

The search strategy we used for our study has been described in detail in another study by Pedersen et al. [24]. The search strategy was a mix of ‘free text words’ (searched in title, abstract and keywords) and ‘defined keywords’ (chosen from the Thesaurus lists of Global Health, Sociological Abstracts, and SPORTdiscus). Appendix 1 presents the entire search strategy.

### Inclusion and exclusion criteria

The inclusion and exclusion criteria were set based on the purpose of this study, and the selected studies for review were required to meet one of the following two criteria:

1. Combining (a) PA in a broad understanding with (b) built environment opportunities for PA and (c) social environment factors (adult or elderly males and/or females aged 15 years or more and social environment factors as defined above).
2. Combining (b) built environment opportunities for PA and (c) social environment factors (adult or elderly males and/or females aged 15 years or older and social environment factors, as defined above).

**Fig. 1:**
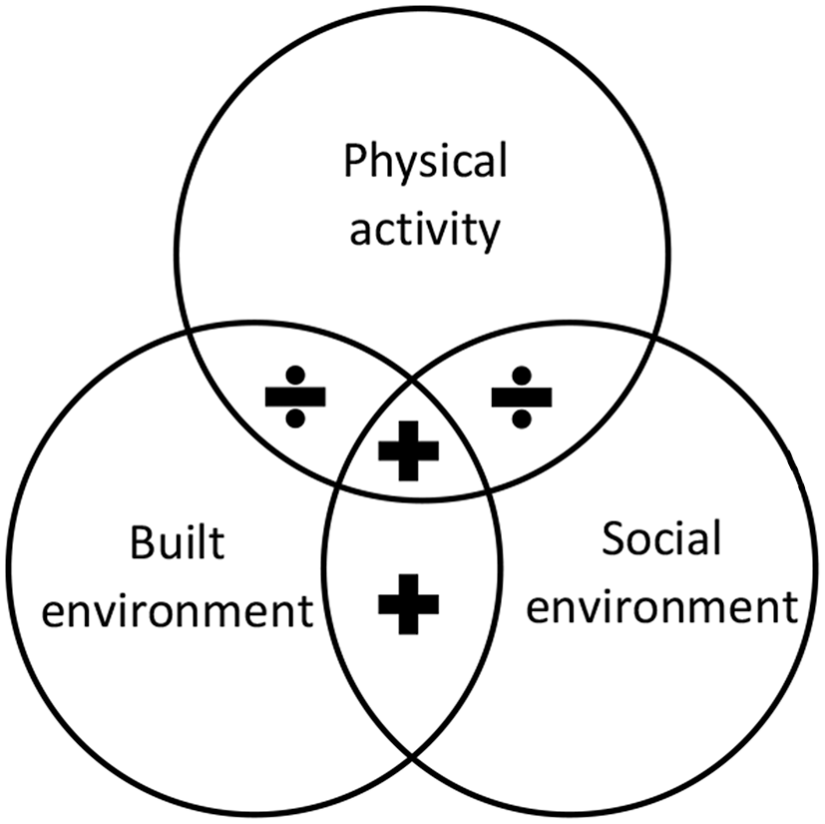
Visual presentation of the inclusion and exclusion criteria. *Footnote* Studies were included within the delineated regions marked by crosses (+)

In Fig. 1, the visual representation regarding this is depicted. Within the delineated regions marked by crosses (+), articles will be included.

Studies were excluded if they focused exclusively on specific types of physical activity (e.g. hang-gliding or parkour). Additionally, research focusing solely on particular ethnic minority groups (e.g. without comparison to the country’s majority population or similar groups) or specific disability groups (e.g. exclusively individuals with visual impairments) was also omitted.

### Screening and selection

**Fig. 2:**
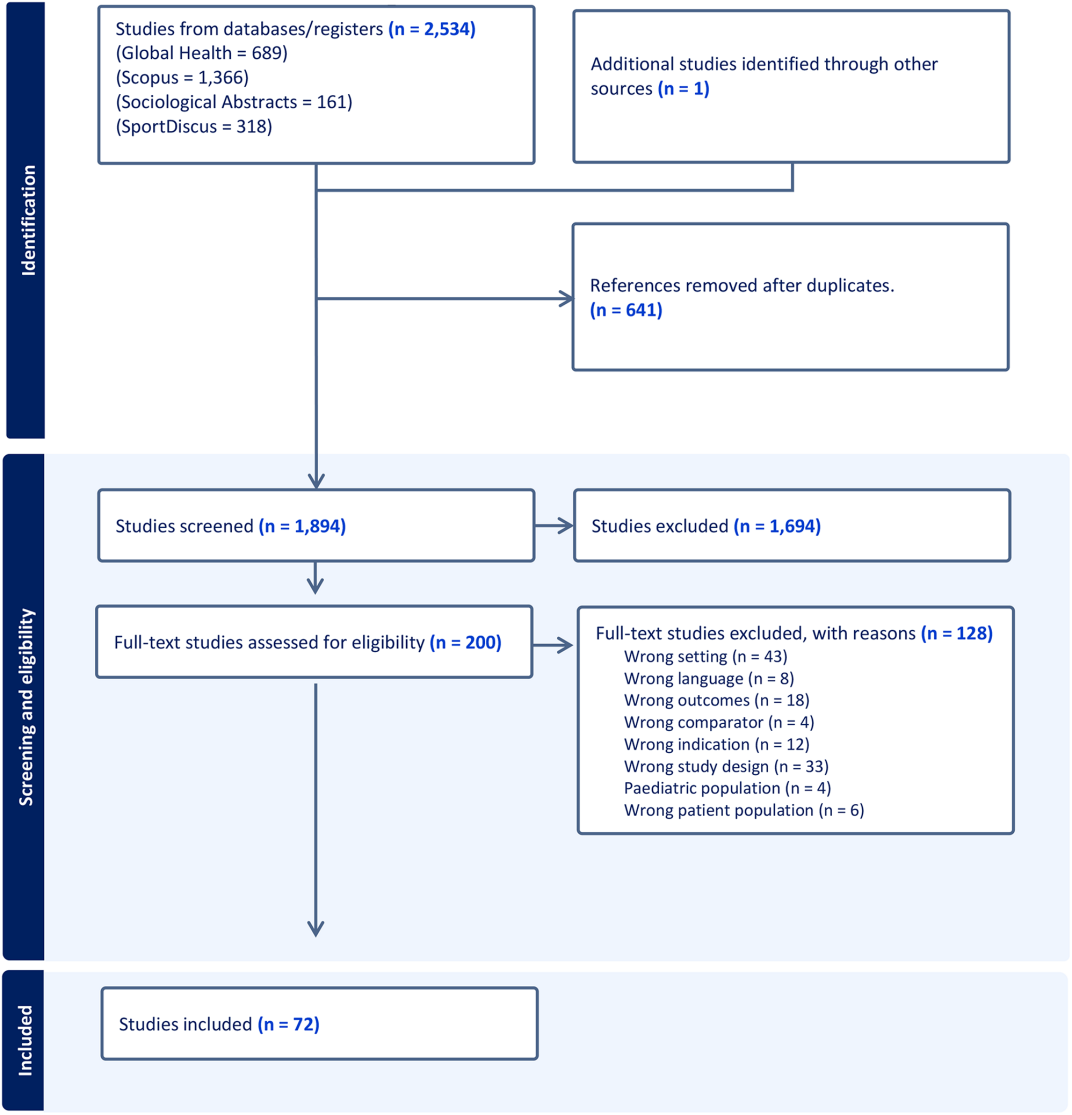
Flow chart of search results

A total of 2,534 references were identified from the four databases. The search results were imported into the library software Endnote, and 641 duplicates were removed. After removing duplicates, 1,894 studies were uploaded to Covidence software (© 2022 Covidence), which was developed for systematic literature reviews. Title and abstract screening full-text screening was performed by two independent screeners (JHK and EBS). First, two authors screened 50 studies to internally validate the screening process. Subsequently, the same two authors screened the rest of the studies. In cases of disagreement, a consensus was reached between the two authors. Reasons for exclusion from the full text have been reported. Studies were selected based on the inclusion and exclusion criteria listed in Sect. 2.4. Through title and abstract screening, 1,694 studies were found to be irrelevant, and 200 studies were assessed as eligible for full-text screening, of which 128 were excluded for reasons (see Fig. 2). 72 studies were included for further analysis and reviewed by two authors. See Fig. 2.

### Data extraction and interpretation of data

A structured spreadsheet was developed to identify common themes for extracting data from all studies included in the review. The data extracted included the year of publication, study location, study population and/or environment, study design, purpose, key findings, and focus related to the four types of built environments (walking infrastructure, cyclist infrastructure, neighbourhood parks, open spaces, and sports facilities) (Appendix 2). Using this spreadsheet, we summarised the data, followed by an interpretation of the patterns and trends shown in the literature. As this was a scoping review [23], formal evidence synthesis was not undertaken. Instead, we conducted a thematic analysis (described in the following section) to identify recurring themes across the studies. Subsequently, we developed a narrative synthesis through consensus meetings to describe, validate, and consolidate common findings and patterns in the results.

## Results

This review included 72 studies dealing with the association between social environment, PA, and opportunities for PA within the four types of built environments. Of these, 28 studies were from European countries, one from South Africa, five from Asia, 16 from North America, three from South America, and nine from Oceania. Studies that include more than one country count of 10 are mainly conducted across European, Oceania, and American countries. The country where we find most studies is England, followed by the USA and Australia (Table 1). The selected studies were published between 2002 and 2022 and included both quantitative and qualitative studies (Appendix 2).

**Table 1:** Descriptive information about the included studies’ origins (continent and country)

Continent and country	No. of studies
**Europe**	
France	2
Germany	1
Netherland	1
Norway	1
Scotland	3
Spain	5
Sweden	2
England	13
**Africa**	
South Africa	1
**Asia**	
China	3
Israel	1
South Korea (ROK)	1
**North America**	
Canada	6
Mexico	1
USA	9
South America	
Brazil	3
**Oceania**	
Australia	9
**More than one country** (1 of these studies contained only European countries, and 1 contained only US and Australian countries)	10

Based on our thematic analysis, all 72 studies were categorised into four types of built environment, as shown in Table 2. Some studies could be categorised into more than one category, which is why the total number of studies was greater than 72.

**Table 2:** The number of studies that examined social environment related to opportunities for PA within each of the four types of built environments

Built environment	No. of studies
Walking infrastructure	33
Cycling infrastructure	9
Neighbourhood parks and open spaces	19
Sports facilities	35
Across all four environments	2

In studies exploring opportunities for PA within the four types of built environments in association with PA and social environments, a range of indices and measures were employed. The measurements of PA encompassed a comprehensive range of methods, including self-reported cross-sectional surveys, objective measures using accelerometers, qualitative interviews, observations, environmental assessments, interventions, and social determinants analysis. Methods employed to assess and measure various aspects of the built environments included walkability scores, geospatial/GIS analyses, audio-visual narratives and park audit tools. Finally, we also included a number of review studies which also spans various methods and measures relating PA.

Below, we provide an overview of the selected studies and their results organised by the four types of built environments. For each built environment, we further divided sections up between results that focus on area and individual specific variables.

### Walking infrastructure (including street and pedestrian connectivity, land use, density, transit proximity and access, aesthetics and design)

#### Results focusing on area variables

When looking into walking infrastructure in the context of societal or area factors of the social environment, and how it is related to opportunities for PA, we found four studies at the community level, indicating that high-SES areas tended to have higher walkability scores than low-SES areas [25–28]. A walkability score is a measure of how conducive an area is to walking and is influenced by the walking infrastructure factors. Having a higher walkability score indicate neighborhoods where walking is more convenient, safe, and enjoyable [20]. Jacobs et al. [29] did, however, find variations across their studies included in their review, with some studies highlighting that areas with higher SES tend to have superior walking infrastructure and greater amount of walking tracks, while other studies find the opposite. The presence and accessibility of walking facilities are generally identified as supporting walking [30, 31]. In contrast to studies suggesting higher walkability scores in high-SES areas, an inverse relationship was found in studies by Choi and Yoon [32] and Conderino et al. [2]. The last study reported that on average, low-income neighbourhoods had higher walking scores than high-income ones. Notably, most white neighbourhoods generally had lower walk scores than other racial/ethnic majority neighbourhoods, except for the majority of black neighbourhoods, where tracts in lower income tertiles had the lowest walkability.

Perceptions within neighbourhoods also affect objective walkability measurements. Higher-income areas are often perceived as more aesthetically pleasing, with higher quality, fewer physical barriers to walking, and lower levels of crime and traffic [33, 34]. Conversely, low-SES areas tend to have poorer perceived built environmental experiences [30, 31, 34]. Giles-Corti and Donovan [30] suggest that the quality of the built walking environment may be more important than the SES of the area of residence, as a correlate of walking behaviour. Other findings is also highlighting the built environmental factors, such as pedestrian bridges over large roads, well-maintain pavements, and illuminated walk-and-bike paths, as encouraging and crucial for walking behaviour [31, 35].

Another aspect related to walking infrastructure opportunities for PA is the density of the area. Remote areas tend to have poorer walking and bicycle infrastructure, lower walkability scores, and less favourable structural attributes for PA [36]. Two studies found that areas with higher intersection density and connectivity, often urban, with multiple destinations and branched road networks, tend to promote walking and meeting PA recommendations [37, 38]. The same result was observed among low-SES adults in a study by Christie et al. [39]. In contrast, Boone-Heinonen and Gordon-Larsen [40] found that higher landscape diversity was associated with higher PA, and for females, higher street connectivity was linked to lower PA. Furthermore, Isiagi, Okop, and Lambert [41] observed a negative association between intersection density and PA regardless of group. Wang et al. [42] also inversely observed a positive association between the built environment and PA in neighbourhoods characterised by low housing density, low road coverage, less land-use diversity (e.g. single land use of residence), high car dependency, poor access to public transport, longer distances to the city, and more green space coverage. Similarly, Frost et al. [43] found positive associations between aesthetics, pathways, safety from crime and traffic, parks, the ease of walking between destinations in the environment, and PA among adults in rural areas.

Furthermore, three studies found that living in high-SES areas is closely related to increased active transportation, higher PA levels, or more steps pr. day [35, 44, 45]. However, Seguin-Fowler et al. [44] found no association between the walk score and PA for those living in low-SES neighbourhoods. Isiagi, Okop, and Lambert [41] inversely found that residents in low-SES/high walkable neighbourhoods reported more transport-related PA compared to high-SES/low walkable neighbourhoods. Similar results were found by Besor et al. [46], who stated that areas characterised by lower-SES residents and a higher proportion of Arab minorities had better-performing health programmes (higher PA). Zang et al. [45] found that the PA of people living in low-SES areas was more dependent on the built environment, whereas the association was limited in high-SES areas. In studies of interventions in both high- and low-SES areas, a positive change in neighbourhood walkability was associated with increased PA, especially in adults in low-SES areas [27, 47, 48]. In a study by Clary et al. [27], improvements in walkability scores were mostly driven by increases in residential density and land-use mix. In contrast, Adkins et al. [49] concluded that the built environment has weaker effects on walking and physical activity in disadvantaged groups than in advantaged ones.

In summary, area-specific studies had different indications. Some studies found varying associations between walking infrastructure factors, there walkability score and PA (including transportation walking) [50, 51], whereas others reported clear associations between higher walkability scores and increased PA across different SES areas [28, 52]. Finally, Hillsdon et al. [53] found that most people engage in PA beyond an 800-metre radius from their homes, suggesting that neighbourhood characteristics alone may not predict PA levels.

#### Results focusing on individual variables

At the individual level, multiple studies have shed light on the interplay between determinants of the social environment, walking infrastructure, and PA. Gullon et al. [54] indicated that individuals with lower income levels tend to have more accessible walking destinations nearby. Furthermore, Christe et al. [47] revealed that the percentage change in walkability scores was positively associated with increased walking, particularly among those with lower income and education levels. Conversely, Cerin and Leslie [33] found that individuals with higher education and income may choose and afford to live in more PA-friendly built environments, including areas conducive for walking. Similarly, Andrade et al. [48] observed that individuals with higher incomes have better access to free or low-cost recreational facilities (including walking trails), a pattern that is also prevalent among those with higher education and more working hours. When examining the use of newly built walking and cycling infrastructure, Smith et al. [55] found that lower educational level and income, rather than ethnicity, were associated with reduced usage.

Dias et al. [56] explored the associations between built environmental factors (objectively and subjectively) and leisure walking among boys and girls with different SES backgrounds. For girls with low SES, access to services and shorter distance to parks and squares were positively associated with leisure walking. For boys, perceived environmental factors such as crime safety, land-use mix, neighbourhood recreation facilities, and places for walking are crucial factors for leisure walking. Another relevant study by Burton et al. [57] revealed that participants across income groups (low, intermediate, and high) place equal importance on similar factors, such as low crime, friendly neighbours, streetlights, and good paths, according to PA. Individuals with higher incomes only marginally emphasised these factors in their PA considerations. Similarly, Cleland et al. [58] found that individual factors, especially those of women with low SES, outweighed environmental factors. Specifically, higher PA levels among low-SES women were associated with interesting local walking opportunities and busy roads to cross during walking.

### Cycling infrastructure (including biking paths, trails, path connectivity and quality)

#### Results focusing on area variables

At the area level, low-SES areas tend to have fewer biking paths compared to their high-SES counterparts [25, 29, 36]. Additionally, Darcy et al. [36] discovered that areas with more disadvantages, often residential areas, within the same local government area have lower quality PA opportunities than less disadvantaged areas. Remote areas also tend to have fewer functional PA opportunities (including walking and bicycle infrastructure) because of poorer structural aspects affecting streets and pathways [36]. The quality of infrastructure, including connected pathways, is considered crucial for transport-biking [31]. In Sweden, shortcomings in structural aspects, quality, and supportive features such as narrow bike paths, inadequate lightning, and concerns about personal safety were found to hinder cycling activity, especially for low-SES citizens [25]. This observation aligns with another Swedish survey study, indicating that active transport to and from school is nearly three times more common among adolescents (16–19 years) living in neighbourhoods with illuminated walking and bike paths than among those without [35]. The same study found that adolescents living in high-SES areas were 80% more likely to bike or walk to school than adolescents living in low-SES areas, and active transportation was 50% less common among adolescents from middle-SES areas than among those in low-SES areas.

#### Results focusing on individual variables

At the individual level, a study conducted in London found that cycling for transportation was more common among white Britain (5.8% vs. 3.0% for ethnic minorities) and people with shorter transportation distances. After accounting for individual and area characteristics, this study also revealed that women and ethnic minorities are less likely to cycle. In contrast to England as a whole, cycling in London became increasingly concentrated among higher-SES groups over time, and increased infrastructure expenditure was associated with more cycling [59]. Similarly, a review by Smith et al. (2017) found in one study that newly built walking and cycling paths were used more by people with higher incomes, higher educational levels, and employment. [55]. Most of these patterns were consistent with Andrade et al. [48], who found that 24% of those with access to free or low-cost recreational facilities (including bicycle infrastructure) had a household income of at least USD 100,000 per year compared to 15.1% of those without access. Similar patterns were observed among those with higher educational levels and working hours.

In summary, a common feature across many studies is that access, length of the bike paths, and quality are associated with physical activity [25, 35, 46, 48]. However, some studies investigating the association between cycle infrastructure, physical activity, and social environment have also found moderators pointing in different directions, leading to no clear conclusions [50, 56].

### Neighbourhood parks and open spaces

#### Results focusing on area variables

Two review studies indicated positive links between PA and neighbourhood parks, open spaces, and general green spaces, potentially reducing socioeconomic PA inequalities [31, 43]. However, Giles et al. [51] presented a contrasting view on the limited benefits of green spaces in low-SES areas, highlighting the complexity of the relationship between green spaces and PA. Doiron et al. [26] observed that high-deprivation neighbourhoods had less access to greenness, affecting PA. Mears et al. [60] showed that residents from deprived areas in Sheffield made shorter, less active visits to green spaces. In contrast, Garrett et al. [61] found that access to green spaces significantly boosts PA through non-recreational activities, such as walking or jogging, particularly for low- and middle-income groups. Zhang et al. [62] underscores the importance of park safety in influencing adolescents’ PA, especially in low-income neighbourhoods, suggesting that perceived safety is a crucial determinant of park utilisation. This is complemented by Sun and Lu [34], who noted significant variations in safety perceptions across income groups affecting park use and the types of activities undertaken. Fontan-Vela et al. [63] and Schneider et al. [64] discussed how residents in higher-SES areas report more park use and fewer barriers, suggesting that these areas might offer better-maintained facilities and safer environments. Conversely, residents in lower SES areas cite limitations such as job constraints, perceived insecurity, and lack of suitable facilities, which hinder their park use and PA engagement. Wang et al. [42] revealed that neighbourhoods with more green spaces in high-SES areas correlate with higher levels of PA, emphasising the role of built environmental quality and accessibility in promoting active lifestyles. However, the proportion of green spaces also tends to be higher in high-SES areas than in low-SES areas, where the distance to and number of green spaces varies across SES areas according to the country in which the studies were conducted [29]. Fontan-Vela et al. [63] reported higher PA in parks within neighbourhoods with high socioeconomic status, citing fewer barriers than in lower-status areas. Schneider et al. [64] found equitable access to parkrun events across deprivation levels in England, but participation from local residents was low, highlighting the need for additional activation measures. Cohen et al. [65] in Los Angeles found that park use in low-income neighbourhoods was gendered, with women’s activities more sedentary compared to men’s. García-Pérez et al. [66] showed that park presence had little influence on women’s leisure-time PA. Finally, Jayasinghe et al. [67] highlighted the challenges in enhancing access to PA infrastructure and natural amenities across socioeconomic disparities.

#### Results focusing on individual variables

A review indicated that SES impacts greenspace use for PA, with complex influences from built environment characteristics. Older adults with a higher SES engage more in PA in neighbourhoods with safe and pleasant built environments and abundant recreational facilities [68]. Anthun et al. [69] found no significant PA changes in a Norwegian suburb over three years, highlighting the importance of location, availability, and social spaces for motivation, with lower SES groups frequently using greenspaces, but dissatisfied with their quality. Clary et al. [28] linked daily moderate-to-vigorous physical activity (MVPA) to the distance to local parks in England, suggesting that travelling to parks boosts PA levels because of limited park facilities. A follow-up study by Clary et al. [27] found no evidence that improved greenspace access affects PA changes across SES groups. Gullon et al. [54] observed that low-income individuals had more green land cover nearby, but might perceive these areas as unsafe for PA, indicating socioeconomic disparities in PA engagement and greenspace perception. This is supported by Compernolle et al. [50], who stated that adults who perceive a greater number of destinations, such as recreational facilities, and those who live in neighbourhoods with more objectively measured aesthetic features, such as trees, green spaces, and parks, are more active.

### Sports facilities

#### Results focusing on area variables

Two review studies initiated a discussion of area-specific results. Jacobs et al. [29] observed varied sports facility access across SES areas in 59 studies with no consistent associations found, whereas Frost et al. [43] identified positive associations between recreational facilities and PA in rural areas. Jayasinghe et al. [67] discovered good sports facility coverage in NW Tasmania, yet this did not lead to high sports participation, suggesting issues with facility visibility or activation. Eime et al. [70] reported a positive association between sports participation and facility availability in Australia adjusted for socioeconomic status and urbanisation, with higher participation in less urbanised regions. Hoekman et al. [71] explored rural-urban differences in sports participation in the Netherlands, highlighting the role of social environment in local sports engagement and the impact of facility diversity. Reimers et al. [72] found that gym availability significantly influenced rural girls’ sports participation in Germany, contrasting with urban girls and boys. Farrell et al. [73] linked the abundance of sports facilities in rural England to reduced physical inactivity, associating facility satisfaction with lower inactivity rates. Kokolakakis et al. [74] identified socio-demographic and economic factors as influencers of sports participation in England, downplaying the role of sports infrastructure in regional disparities. Billaudeau et al. [75] and Cereijo et al. [76] investigated the accessibility and quality of sports facilities in Paris and Madrid, finding mixed associations between SES and facility availability. Spanish studies by Pascual et al. [14, 77] linked local economic resources with the number of sports facilities and PA, especially among older individuals and women. Hillsdon et al. [78] and studies from Asia [32, 34] observed a positive association between SES and leisure amenity availability. Ferguson et al. [79] and Lamb et al. [80] showed that public transport access in low-income areas provides closer proximity to sports facilities, a difference nullified by car ownership. Panter et al. [81] and Hillsdon et al. [53] discussed how poor facility coverage in deprived English areas affects PA levels, with individuals often travelling beyond local areas for activity. Findings from Canada [82] and a review [38] indicate that women are more sensitive to local conditions and proximity to facilities. Australian research [18, 33] has highlighted disparities in perceived access to sports facilities by income area, with psychosocial factors influencing PA more than built environmental factors. Pascual et al. [83] and Karusisi et al. [84] emphasised socioeconomic factors’ dominance over spatial in sports facility usage, with Boone-Heinonen and Gordon-Larsen [40] noting the impact of varied built environments and safety on young adults’ PA, affected by gender and urban density.

#### Results focusing on individual variables

This section delves into how individual attributes such as age, gender, and socio-economic status influence sports facility utilisation, with Jacobs et al. [29] and Lee et al. [85] noting geographical and socio-demographic variations in access. Liu et al. [86] report lower SES and older individuals are less active in facility usage, highlighting complex factors behind participation. Ellaway et al. [87] found no significant link between sports facility accessibility and activity levels, factoring in SES and urbanization. Bergmann et al. [88] noted women and lower-income individuals in the South Region of Brazil frequently use outdoor gyms, suggesting mitigation of PA disparities. Gardam et al. [89] found that outdoor PA equipment in lower-income areas could reduce access disparities. Cutumisu and Spence [8] showed that objective access and personal factors, such as self-efficacy, impact PA adherence, with subjective perceptions of access not correlating with participation. Compernolle et al. [50] indicated that adults perceiving more neighbourhood destinations are less sedentary. Rovniak et al. [90] identified an ‘Active Leisure’ cluster, showing recreational facility availability boosts leisure-time PA. This is supported by Werneck et al. [91], who found that the presence of public PA facilities near a household was associated with higher leisure-time PA among all quintiles of income and educational level. Burton et al. [57] linked active lifestyles with social support, fewer activity barriers, and health issues among higher-income participants. Langøien et al. [92] highlighted the built environmental impact on PA for minority groups in Europe, emphasising the need for available, appropriate, and culturally sensitive facilities. Studies advocate comprehensive environmental improvements and increased PA knowledge and skills. An English programme providing free access to sports facilities, along with marketing and courses, significantly boosted gym and swim participation, particularly in disadvantaged groups [93].

Table 3 is a result from our narrative synthesis and summarises and integrate our research findings on the interplay between PA, the social environment, and opportunities for PA across four types of built environments: walking infrastructure, cycling infrastructure, neighbourhood parks and open spaces, and sports facilities. In synthesizing the findings of 72 studies, this narrative synthesis highlights the most typical results, focusing on the common themes and patterns that emerged across the built environments. By distilling these studies into a cohesive summary, we provide a comprehensive overview of the main trends and outcomes. However, due to the broad scope and the necessity to concentrate on overarching themes, some nuanced details and specific variations within individual studies are not fully represented, meaning that there will be studies in each of the built environments that can show contradictory results.

**Table 3:** Summary of results for each of the four types of built environment

Environment	Area-specific results	Individual-specific results
Walking infrastructure	- High-SES areas generally show higher walkability scores and PA. - Built environmental quality, such as pedestrian infrastructure, impacts walkability and is typically perceived more pleasant in higher-income areas. - Urban areas with higher intersection density and connectivity tend to promote walking, while remote areas often lack adequate infrastructure for PA. - Studies present mixed findings on walkability scores across SES areas.	- Lower income and education levels correlate with less usage of walkable areas. - Higher-income individuals tend to have better walking infrastructure opportunities. - Perceptions of safety and aesthetics influence walking behaviour, and often play more importance than the built environment.
Cycling Infrastructure	- Generally fewer biking paths in low-SES areas. - Quality and connectivity of cycling infrastructure vary by area-SES; however, it tends to be best in high-SES areas.	- Cycling for transport tends to be more common in higher SES groups. - Individuals with higher income and education levels, are more likely to have access to cycling infrastructure. - Infrastructure expenditure is typically linked to increased cycling.
Neighbourhood Parks and Open Spaces	- Several positive associations between access to green spaces and PA, with variations by SES. - Safety and accessibility of parks influence utilization, especially in low-income areas.	- Access to green spaces seems to boosts PA, especially in lower SES groups. - Safety perceptions crucial for park use. - The perception of opportunities for PA in green spaces are crucial for PA.
Sports Facilities	- Varied access to sports facilities by SES, with rural areas often having better access. - Quality and availability of facilities tends to impact PA engagement. - Disparities in perceived access to sports facilities exist based on income areas, with social factors often influencing PA more than built environmental factors.	- Social support and fewer barriers to PA noted among higher-income participants. - Lower SES and education levels is often associated with reduced facility usage. - Objective access and personal factors impact PA adherence, with subjective perceptions of access not always correlating with participation.

## Discussion

This scoping review examines the intricate association between the social environment and opportunities for PA within the built environment and supports our study’s initial assertion that both factors significantly influence PA levels. The diverse outcomes observed in relation to walking infrastructure, cycling infrastructure, neighbourhood parks, open spaces, and sports facilities emphasise the intricacy of these relationships. Our study’s dual focus on area- and individual-specific influences, as outlined in Table 3, offers a distinct perspective for understanding how both social and built environmental attributes function as critical facilitators or barriers to access to PA opportunities and engagement in PA.

Given the global imperative to combat sedentary lifestyles and their associated health risks, our discussion delves into the implications of our findings in a broader context of health promotion. Moreover, we address the notable disparities in PA opportunities and engagement across social environment variables, underscoring the importance of targeted interventions that are sensitive to both the built environment and individual determinants of PA.

### Area-specific and individual-specific influences on PA engagement

As we dissect the implications of our findings, it becomes increasingly evident that the determinants of PA are not monolithic but rather a tapestry of intertwined area-specific and individual-specific factors. First, area-specific results revealed the profound impact of the built environment on PA opportunities. Walking and cycling infrastructure and the availability of parks and sports facilities do not uniformly benefit all community members. Instead, their influence is modulated by the socioeconomic fabric of neighbourhoods, revealing a gradient of accessibility that mirrors societal inequities.

However, several studies in our review also investigated the subjective perceptions of accessibility, connectivity, and built environment quality [56, 58], which made it clear that the built environment’s impact on individual behaviour cannot be fully explained by objectively measured indicators. The individual-specific results illuminate the equally pivotal role of personal factors, from socioeconomic status to perceptions of safety and aesthetics, in shaping PA behaviour. These findings underscore the subjective nature of PA engagement and the understanding of opportunities for PA, in which personal motivations, perceptions, and barriers play a role against the backdrop of available environmental resources, which is also supported by the findings of Wang et al. [15], (2023).

The mutual dependency between personal factors and physical environment underscores the need for a comprehensive strategy to advance PA. This strategy should encompass both concrete and abstract factors that shape a person’s willingness to engage in PA. These factors align with those outlined in socioecological models, which include policy and environment (e.g. neighbourhood safety), sociocultural factors (e.g. community support for physical activity), and personal beliefs (e.g. perceptions of physical activity) [16]. Such factors are pivotal in shaping socioeconomic disparities in PA behaviour. In particular, the impact of safety perceptions and aesthetic appeal on walking activities underscores their critical role, often surpassing the influence of the physical setting on PA engagement.

### Addressing social environment disparities

The disparities in physical activity underscored by our findings highlight the complex interplay between social environmental factors and access to PA-enhancing environments. Area-specific variables revealed a clear contrast in the availability and quality of walking and cycling infrastructure, neighbourhood parks, open spaces, and sports facilities across high- and low-SES areas. Our analysis indicates that high-SES areas typically enjoy superior walking infrastructure, more extensive and better-maintained cycling infrastructure, and greater access to parks and sports facilities. This built environmental privilege translates into higher levels of PA among residents, underscoring the need to shift towards built environmental equity. Urban planning and policies must prioritise the development and maintenance of PA infrastructure in low-SES areas, ensuring that all community members have equal opportunities to engage in health promoting physical activities.

Our results on the individual-specific variables confirm this association, as we find that individuals with low income and educational levels have less access to facility opportunities for PA across the four types of built environments. At the same time, it also highlights how lower income and education levels mostly correlate with reduced utilisation of opportunities for PA within the built environment. Most often, it is also the lower SES groups that have poorer perceptions of their opportunities for PA, their safety, aesthetics, and availability, pointing towards a multifaceted challenge that requires nuanced solutions. The influence of individual-specific variables on PA participation and PA opportunities thus cannot be overstated. Consequently, interventions aimed at increasing PA and opportunities must address these perceptions directly. Community engagement initiatives involving residents in the planning and maintenance of PA facilities can enhance the sense of ownership and safety. Moreover, programmes designed to boost social support for PA within communities can help overcome individual barriers and encourage more residents to lead more active lifestyles; physical features such as lightning and aesthetics can improve the perception of opportunities to engage in PA within the local environment, especially in low-SES areas.

### Suggestions for future research

This review indicates that many researchers have focused on the relationship between social and built environments for PA; however, more literature focusing on individual perceptions of opportunities for PA within the built environment across different social environment indicators is needed. Further exploration of the subjective perceptions of accessibility, aesthetics, connectivity, and built environment quality across different social environment indicators (individual- and area-specific factors) can provide deeper insights into how individuals perceive their environment, how these perceptions influence their PA behaviours, and how an understanding of how multiple factors intersect to shape PA behaviours can inform more targeted interventions.

### Strengths and limitations

The strengths of this study include a comprehensive analysis of the interaction between the determinants of the social environment and opportunities for PA within built environments, highlighting the importance of equitable access to recreational facilities. This underscores the role of urban planning in promoting health through infrastructure. However, it faces limitations, such as potential biases in self-reported physical activity data, lack of longitudinal data to establish causality, absence of higher-quality systematic reviews articles included on the theme, and possible oversimplification of the complex interplay between socioeconomic factors and physical activity behaviours.

Despite the potential to advance our understanding of global health disparities, when investigating the interplay between the social environment and opportunities for physical activity across countries, we must recognise that there are limitations that should be considered. When comparing results across countries, there can be various cultural norms and values regarding PA. For example, what constitutes acceptable or accessible forms of activity can vary greatly, influencing how the determinants of the social environment interact with built environments to shape physical activity opportunities. Furthermore, differences regarding the socioeconomic context, urban planning disparities, data availability and quality, policies, environmental context, and the like, can make it challenging to generate meaningful insights into the complex relationship between social- and built environments, and physical activity outcomes on a global scale. This could be differences related to density and diversity across countries in relation to SES-factors. In the US, higher SES, for example, often correlates with suburban areas that have lower walkability scores due to less density and diversity. These areas tend to have higher levels of PA despite the lower walkability scores, possibly due to greater access to private facilities and transportation, where in European studies, this association might be different and show other patterns.

## Conclusions

This review illuminates the complex interplay between social and built environments that affects opportunities for physical activity (PA) and the impact on PA levels. This highlights the significant role of socioeconomic factors and the quality of PA infrastructure in promoting or hindering PA across communities. Notably, disparities in access to PA resources underscore the need for equitable urban planning and public health interventions.

This study’s insights are crucial for developing targeted strategies that address both physical and social barriers to PA. Advocating inclusive and accessible PA facilities calls for a unified approach to enhance PA levels universally, emphasising the importance of addressing socioeconomic disparities in PA access.

This review advocates integrated efforts to ensure equitable access to PA opportunities, aiming to support health and well-being for all, regardless of socioeconomic status. These findings are vital for informing more effective public health policies and urban planning strategies that foster a more active and healthier society.

### Electronic supplementary material

Below is the link to the electronic supplementary material.

Supplementary Material 1

Supplementary Material 2

## Data Availability

All data have been published as supplemental materials and collected from published articles.

## Abbreviations

PA: Physical activity
SES: Socioeconomic status

## References

[1] World Health Organization. WHO. Global status report on physical activity 2022. Geneva: World Health Organization; 2022.

[2] Conderino SE, Feldman JM, Spoer B, Gourevitch MN, Thorpe LE. Social and economic differences in neighborhood walkability across 500 U.S. cities. Am J Prev Med. 2021;61:394–401. 34108111 10.1016/j.amepre.2021.03.014

[3] Davison KK, Lawson CT. Do attributes in the physical environment influence children’s physical activity? A review of the literature. IntJBehavNutrPhysAct. 2006;3:19. 10.1186/1479-5868-3-19 PMC1557665 16872543

[4] National Center for Chronic Disease Prevention and Health, Promotion C. The built environment. An Assessment Tool and Manual. (an adaptation of MAPS). Atlanta, USA: National Center for Chronic Disease Prevention and Health Promotion. Division of Community Health; 2015.

[5] Bauman AE, Bull F. Environmental correlates of physical activity and walking in adults and children: a review of reviews. NICE Bull. 2007;44.

[6] Badland H, Schofield G. Transport, urban design, and physical activity: an evidence-based update. Transp Res Part Transp Environ. 2005;10:177–96. 10.1016/j.trd.2004.12.001

[7] Saelens BE, Handy SL. Built Environment correlates of walking: a review. Medicine and science in sports and exercise. Hagerstown, MD: Lippincott Williams & Wilkins; 2008. pp. S550–66. 10.1249/MSS.0b013e31817c67a4 PMC2921187 18562973

[8] Cutumisu N, Spence JC. Sport fields as potential catalysts for physical activity in the neighbourhood. IntJEnvironResPublic Health. 2012;9:294–314. 10.3390/ijerph9010294 PMC3315071 22470293

[9] Pharr JR, Lough NL, Terencio AM. Sociodemographic determinants of Physical Activity and Sport participation among women in the United States. Sports Basel. 2020;8:96. 32630832 10.3390/sports8070096 PMC7404454

[10] Shuval K, Li Q, Gabriel KP, Tchernis R. Income, physical activity, sedentary behavior, and the ‘weekend warrior’ among U.S. adults. Prev Med. 2017;103 Journal Article:91–7. 10.1016/j.ypmed.2017.07.033 28802654

[11] Scheers T, Philippaerts R, Lefevre J. Compliance with different physical activity recommendations and its association with socio-demographic characteristics using an objective measure. BMC Public Health. 2013;13:136–136. 23409982 10.1186/1471-2458-13-136 PMC3599794

[12] Blazer DG, Hernandez LM, Genes. Behavior, and the Social Environment: moving beyond the Nature/Nurture debate. In: Hernandez LM, Blazer DG, editors. Genes, behavior, and the social environment. USA: National Academies.; 2006. pp. 25–43. 20669442

[13] Kim Y, Ritchie L, Landgraf A, Hasson RE, Colabianchi N. The role of the Neighborhood Social Environment in physical activity among hispanic children: moderation by Cultural factors and mediation by Neighborhood norms. Int J Environ Res Public Health. 2020;17:9527. 33352648 10.3390/ijerph17249527 PMC7766550

[14] Pascual C, Regidor E, Astasio P, Ortega P, Navarro P, Domínguez V. The association of current and sustained area-based adverse socioeconomic environment with physical inactivity. Soc Sci Med. 2007;65:454–66. 17466424 10.1016/j.socscimed.2007.03.023

[15] Wang Y, Steenbergen B, van der Krabben E, Kooij H-J, Raaphorst K, Hoekman R. The impact of the built Environment and Social Environment on physical activity: a scoping review. Int J Environ Res Public Health. 2023;20:6189. 37372774 10.3390/ijerph20126189 PMC10297989

[16] Gubbels JS, Van Kann DH, de Vries NK, Thijs C, Kremers SP. Next step in health behavior research: the need for ecological moderation analyses - an application to diet and physical activity at childcare. Int J Behav Nutr Phys Act. 2014;11:52–52. 24742167 10.1186/1479-5868-11-52 PMC4002539

[17] Christian H, Giles-Corti B, Knuiman M, Timperio A, Foster S. The influence of the built environment, social environment and health behaviors on body mass index. Results from RESIDE. Prev Med. 2011;53:57–60. 21609730 10.1016/j.ypmed.2011.05.004

[18] Giles-Corti B, Donovan RJ. The relative influence of individual, social and physical environment determinants of physical activity. Soc Sci Med 1982. 2002;54:1793–812. 10.1016/s0277-9536(01)00150-2 12113436

[19] Brug J, Oenema A, Ferreira I. Theory, evidence and intervention mapping to improve behavior nutrition and physical activity interventions. Int J Behav Nutr Phys Act. 2005;2. 10.1186/1479-5868-2-2 PMC1087867 15807898

[20] McCormack G, Shiell A. In search of causality: a systematic review of the relationship between the built environment and physical activity among adults. Int J Behav Nutr Phys Act. 2011;8:125. 22077952 10.1186/1479-5868-8-125 PMC3306205

[21] Caspersen CJ, Powell KE, Christenson GM. Physical activity, Exercise, and physical fitness: definitions and distinctions for Health-Related Research. Public Health Rep 1974. 1985;100:126–31. PMC1424733 3920711

[22] Pedersen MR, Bredahl TV, Elmose-Østerlund K, Hansen AF. Motives and barriers related to physical activity within different types of built environments: implications for Health Promotion. Int J Environ Res Public Health. 2022;19. 10.3390/ijerph19159000 PMC9330905 35897374

[23] Peters MD, Godfrey CM, Khalil H, McInerney P, Parker D, Soares CB. Guidance for conducting systematic scoping reviews. Int J Evid Based Heal. 2015;13:141–6. 10.1097/XEB.0000000000000050 26134548

[24] Pedersen MRL, Hansen AF, Elmose-Østerlund K. Motives and barriers related to physical activity and Sport across Social backgrounds: implications for Health Promotion. Int J Environ Res Public Health. 2021;18:5810. 34071630 10.3390/ijerph18115810 PMC8198157

[25] Rydenstam T, Fell T, Buli BG, King AC, Bälter K. Using citizen science to understand the prerequisites for physical activity among adolescents in low socioeconomic status neighborhoods - the NESLA study. Health Place. 2020;65. 10.1016/j.healthplace.2020.102387 32889390

[26] Doiron D, Setton EM, Shairsingh K, Brauer M, Hystad P, Ross NA et al. Healthy built environment: spatial patterns and relationships of multiple exposures and deprivation in Toronto, Montreal and Vancouver. Environ Int. 2020;143. 10.1016/j.envint.2020.106003 32763633

[27] Clary C, Lewis D, Limb E, Nightingale CM, Ram B, Page AS et al. Longitudinal impact of changes in the residential built environment on physical activity: findings from the ENABLE London cohort study. Int J Behav Nutr Phys Act. 2020;17. 10.1186/s12966-020-01003-9 PMC7395376 32738916

[28] Clary C, Lewis D, Limb ES, Nightingale CM, Ram B, Rudnicka AR et al. Weekend and weekday associations between the residential built environment and physical activity: findings from the ENABLE London study. PLoS ONE. 2020;15 9 September. 10.1371/journal.pone.0237323 PMC7467308 32877423

[29] Jacobs J, Alston L, Needham C, Backholer K, Strugnell C, Allender S, et al. Variation in the physical activity environment according to area-level socio-economic position—A systematic review. Obes Rev. 2019;20:686–700. 30624854 10.1111/obr.12818

[30] Giles-Corti B, Donovan RJ. Socioeconomic status differences in recreational physical activity levels and real and perceived access to a supportive physical environment. PrevMed. 2002;35:601–11. 10.1006/pmed.2002.1115 12460528

[31] Salvo G, Lashewicz BM, Doyle-Baker PK, McCormack GR. Neighbourhood built environment influences on physical activity among adults: a systematized review of qualitative evidence. Int J Environ Res Public Health. 2018;15. 10.3390/ijerph15050897 PMC5981936 29724048

[32] Choi Y, Yoon H. Do the walkability and urban leisure amenities of neighborhoods affect the body mass index of individuals? Based on a case study in Seoul, South Korea. Int J Environ Res Public Health. 2020;17. 10.3390/ijerph17062060 PMC7142730 32244911

[33] Cerin E, Leslie E. How socio-economic status contributes to participation in leisure-time physical activity. Soc Sci Med. 2008;66:2596–609. 18359137 10.1016/j.socscimed.2008.02.012

[34] Sun P, Lu W. Environmental inequity in hilly neighborhood using multi-source data from a health promotion view. Environ Res. 2022;204 Part A. 10.1016/j.envres.2021.111983 34506785

[35] Buli BG, Tillander A, Fell T, Bälter K. Active commuting and healthy behavior among adolescents in neighborhoods with varying socioeconomic status: the NESLA study. Int J Environ Res Public Health. 2022;19. 10.3390/ijerph19073784 PMC8997619 35409464

[36] Darcy M, Parkinson J, McDonald N, Moriarty S, Kadariya S, Sapkota D. Geographic remoteness and socioeconomic disadvantage reduce the supportiveness of food and physical activity environments in Australia. Aust N Z J Public Health. 2022;46:346–53. 35357735 10.1111/1753-6405.13227

[37] Mayne DJ, Morgan GG, Jalaludin BB, Bauman AE. The contribution of area-level walkability to geographic variation in physical activity: a spatial analysis of 95,837 participants from the 45 and up Study living in Sydney, Australia. Popul Health Metr. 2017;15. 10.1186/s12963-017-0149-x PMC5627488 28974226

[38] Prince SA, Reed JL, Martinello N, Adamo KB, Fodor JG, Hiremath S, et al. Why are adult women physically active? A systematic review of prospective cohort studies to identify intrapersonal, social environmental and physical environmental determinants. Obes Rev. 2016;17:919–44. 27465602 10.1111/obr.12432

[39] Christie CD, Consoli A, Ronksley PE, Vena JE, Friedenreich CM, McCormack GR. Associations between the built environment and physical activity among adults with low socio-economic status in Canada: a systematic review. Can J Public Health. 2021;112:152–65. 32833139 10.17269/s41997-020-00364-9 PMC7851286

[40] Boone-Heinonen J, Gordon-Larsen P. Life stage and sex specificity in relationships between the built and socioeconomic environments and physical activity. J Epidemiol Community Health. 2011;65:847–52. 20930092 10.1136/jech.2009.105064 PMC3059385

[41] Isiagi M, Okop KJ, Lambert EV. The relationship between physical activity and the objectively-measured built environment in low-and high-income South African communities. Int J Environ Res Public Health. 2021;18. 10.3390/ijerph18083853 PMC8067549 33916926

[42] Wang S, Liu Y, Lam J, Kwan MP. The effects of the built environment on the general health, physical activity and obesity of adults in Queensland, Australia. Spat Spatio-Temporal Epidemiol. 2021;39. 10.1016/j.sste.2021.100456 34774262

[43] Frost SS, Goins RT, Hunter RH, Hooker SP, Bryant LL, Kruger J, et al. Effects of the built environment on physical activity of adults living in rural settings. Am J Health Promot. 2010;24:267–83. 20232609 10.4278/ajhp.08040532

[44] Seguin-Fowler RA, LaCroix AZ, LaMonte MJ, Liu J, Maddock JE, Rethorst CD et al. Association of neighborhood walk score with accelerometer-measured physical activity varies by neighborhood socioeconomic status in older women. Prev Med Rep. 2022;29. 10.1016/j.pmedr.2022.101931 PMC9502671 36161128

[45] Zang P, Xian F, Qiu H, Ma S, Guo H, Wang M et al. Differences in the correlation between the built environment and walking, moderate, and vigorous physical activity among the Elderly in Low-and high-income areas: is higher income more relevant? Int J Environ Res Public Health. 2022;19. 10.3390/ijerph19031894 PMC8834696 35162915

[46] Besor O, Paltiel O, Manor O, Donchin M, Rauch O, Kaufman-Shriqui V. Associations between density and quality of health promotion programmes and built environment features across Jerusalem. Eur J Public Health. 2021;31:1190–6. 34568902 10.1093/eurpub/ckab132

[47] Christie CD, Friedenreich CM, Vena JE, Turley L, McCormack GR. Cross-sectional and longitudinal associations between the built environment and walking: effect modification by socioeconomic status. BMC Public Health. 2022;22. 10.1186/s12889-022-13611-0 PMC9210749 35729509

[48] Andrade L, Geffin R, Maguire M, Rodriguez P, Castro G, Alkhatib A et al. The associations between Access to Recreational Facilities and Adherence to the American Heart Association’s physical activity guidelines in US adults. Front Public Health. 2021;9. 10.3389/fpubh.2021.660624 PMC8654348 34900883

[49] Adkins A, Makarewicz C, Scanze M, Ingram M, Luhr G. Contextualizing walkability: do relationships between built environments and walking Vary by Socioeconomic Context? J Am Plann Assoc. 2017;83:296–314. 31762526 10.1080/01944363.2017.1322527 PMC6873812

[50] Compernolle S, De Cocker K, Roda C, Oppert JM, Mackenbach JD, Lakerveld J et al. Physical environmental correlates of domain-SpecificSedentary behaviours across five European regions (the SPOTLIGHT Project). PLoS ONE. 2016;11. 10.1371/journal.pone.0164812 PMC5065139 27741310

[51] Giles LV, Koehle MS, Saelens BE, Sbihi H, Carlsten C. When physical activity meets the physical environment: precision health insights from the intersection. Environ Health Prev Med. 2021;26:1–10. 34193051 10.1186/s12199-021-00990-w PMC8247190

[52] Todd M, Adams MA, Kurka J, Conway TL, Cain KL, Buman MP, et al. GIS-measured walkability, transit, and recreation environments in relation to older adults’ physical activity: a latent profile analysis. Prev Med. 2016;93:57–63. 27663428 10.1016/j.ypmed.2016.09.019 PMC5718370

[53] Hillsdon M, Coombes E, Griew P, Jones A. An assessment of the relevance of the home neighbourhood for understanding environmental influences on physical activity: how far from home do people roam? Int J Behav Nutr Phys Act. 2015;12. 10.1186/s12966-015-0260-y PMC4537551 26277369

[54] Gullon P, Bilal U, Hirsch JA, Rundle AG, Judd S, Safford MM, et al. Does a physical activity supportive environment ameliorate or exacerbate socioeconomic inequities in incident coronary heart disease? J Epidemiol Community Health. 2021;75:637–42. 33318134 10.1136/jech-2020-215239 PMC8200362

[55] Smith M, Hosking J, Woodward A, Witten K, MacMillan A, Field A et al. Systematic literature review of built environment effects on physical activity and active transport - an update and new findings on health equity. Int J Behav Nutr Phys Act. 2017;14. 10.1186/s12966-017-0613-9 PMC5693449 29145884

[56] Dias AF, Gaya AR, Santos MP, Brand C, Pizarro AN, Fochesatto CF et al. Neighborhood environmental factors associated with leisure walking in adolescents. Rev Saude Publica. 2020;54. 10.11606/s1518-8787.2020054002222 PMC7263801 32491115

[57] Burton NW, Turrell G, Oldenburg B. Participation in recreational physical activity: why do socioeconomic groups differ? Health Educ Behav. 2003;30:225–44. 12693525 10.1177/1090198102251036

[58] Cleland VJ, Ball K, Salmon J, Timperio AF, Crawford DA. Personal, social and environmental correlates of resilience to physical inactivity among women from socio-economically disadvantaged backgrounds. Health Educ Res. 2010;25:268–81. 18974098 10.1093/her/cyn054

[59] Martin A, Morciano M, Suhrcke M. Determinants of bicycle commuting and the effect of bicycle infrastructure investment in London: evidence from UK census microdata. Econ Hum Biol. 2021;41. 10.1016/j.ehb.2020.100945 33401067

[60] Mears M, Brindley P, Barrows P, Richardson M, Maheswaran R. Mapping urban greenspace use from mobile phone GPS data. PLoS ONE. 2021;16 7 July. 10.1371/journal.pone.0248622 PMC8262795 34232961

[61] Garrett JK, White MP, Elliott LR, Wheeler BW, Fleming LE. Urban nature and physical activity: investigating associations using self-reported and accelerometer data and the role of household income. Environ Res. 2020;190. 10.1016/j.envres.2020.109899 32750550

[62] Zhang R, Zhang CQ, Lai PC, Cheng W, Schüz B, Kwan MP. Park environment and moderate-to-vigorous physical activity in parks among adolescents in a high-density city: the moderating role of neighbourhood income. Int J Health Geogr. 2021;20. 10.1186/s12942-021-00289-7 PMC8365917 34399765

[63] Fontán-Vela M, Rivera-Navarro J, Gullón P, Díez J, Anguelovski I, Franco M. Active use and perceptions of parks as urban assets for physical activity: a mixed-methods study. Health Place. 2021;71. 10.1016/j.healthplace.2021.102660 34454253

[64] Schneider PP, Smith RA, Bullas AM, Quirk H, Bayley T, Haake SJ, et al. Multiple deprivation and geographic distance to community physical activity events — achieving equitable access to parkrun in England. Public Health. 2020;189:48–53. 33157459 10.1016/j.puhe.2020.09.002 PMC7762722

[65] Cohen DA, Han B, Park S, Williamson S, Derose KP. Park use and park-based physical activity in low-income neighborhoods. J Aging Phys Act. 2019;27:334–42. 30160585 10.1123/japa.2018-0032 PMC7494055

[66] García-Pérez H, Lara-Valencia F. Association between Neighborhood Parks and Leisure-time physical activity among adult Mexican women. / Asociación entre parques de barrio y actividad física recreativa en mujeres mexicanas adultas. Retos Nuevas Perspect Educ Física Deporte Recreación. 2021;:544–54.

[67] Jayasinghe S, Flies EJ, Soward R, Kendal D, Kilpatrick M, Holloway TP et al. A spatial analysis of Access to Physical Activity infrastructure and Healthy Food in Regional Tasmania. Front Public Health. 2021;9. 10.3389/fpubh.2021.773609 PMC8671161 34926390

[68] Spencer LH, Lynch M, Lawrence CL, Edwards RT. A scoping review of how income affects accessing local green space to engage in outdoor physical activity to improve well-being: implications for post-COVID-19. Int J Environ Res Public Health. 2020;17. 10.3390/ijerph17249313 PMC7764517 33322829

[69] Anthun KS, Maass REK, Hope S, Espnes GA, Bell R, Khan M et al. Addressing inequity: evaluation of an intervention to improve accessibility and quality of a green space. Int J Environ Res Public Health. 2019;16. 10.3390/ijerph16245015 PMC6950353 31835473

[70] Eime RM, Harvey J, Charity MJ, Casey M, Westerbeek H, Payne WR. The relationship of sport participation to provision of sports facilities and socioeconomic status: a geographical analysis. Aust N Z J Public Health. 2017;41:248–55. 28110514 10.1111/1753-6405.12647

[71] Hoekman R, Breedveld K, Kraaykamp G. Sport participation and the social and physical environment: explaining differences between urban and rural areas in the Netherlands. Leis Stud. 2017;36:357–70.

[72] Reimers AK, Wagner M, Alvanides S, Steinmayr A, Reiner M, Schmidt S, et al. Proximity to Sports Facilities and sports participation for adolescents in Germany. PLoS ONE. 2014;9:e93059. 24675689 10.1371/journal.pone.0093059 PMC3968093

[73] Farrell L, Hollingsworth B, Propper C, Shields MA. The socioeconomic gradient in physical inactivity: evidence from one million adults in England. Soc Sci Med. 2014;123:55–63. 25462605 10.1016/j.socscimed.2014.10.039

[74] Kokolakakis T, Lera-López F, Castellanos P. Regional differences in sports participation: the case of local authorities in England. Int J Sport Finance. 2014;9:149–71.

[75] Billaudeau N, Oppert JM, Simon C, Charreire H, Casey R, Salze P et al. Investigating disparities in spatial accessibility to and characteristics of sport facilities: Direction, strength, and spatial scale of associations with area income. Health Place. 2010;17. 10.1016/j.healthplace.2010.09.004 20870447

[76] Cereijo L, Gullón P, Cebrecos A, Bilal U, Santacruz JA, Badland H et al. Access to and availability of exercise facilities in Madrid: an equity perspective. Int J Health Geogr. 2019;18:(2 July 2019). 10.1186/s12942-019-0179-7 PMC6604462 31266518

[77] Pascual C, Regidor E, Arco DA, Alejos B, Santos JM, Calle ME, et al. Sports facilities in Madrid explain the relationship between neighbourhood economic context and physical inactivity in older people, but not in younger adults: a case study. J Epidemiol Community Health. 2013;67:788–94. 23794611 10.1136/jech-2013-202583

[78] Hillsdon M, Panter J, Foster C, Jones A. Equitable Access to Exercise facilities. Am J Prev Med. 2007;32:506–8. 17533066 10.1016/j.amepre.2007.02.018

[79] Ferguson NS, Lamb KE, Wang Y, Ogilvie D, Ellaway A. Access to recreational physical activities by Car and bus: an Assessment of Socio-spatial inequalities in Mainland Scotland. PLoS ONE. 2013;8. 10.1371/journal.pone.0055638 PMC3567099 23409012

[80] Lamb KE, Ogilvie D, Ferguson NS, Murray J, Wang Y, Ellaway A. Sociospatial distribution of access to facilities for moderate and vigorous intensity physical activity in Scotland by different modes of transport. Int J Behav Nutr Phys Act. 2012;9:55. 22568969 10.1186/1479-5868-9-55 PMC3388950

[81] Panter J, Jones A, Hillsdon M. Equity of access to physical activity facilities in an English city. Prev Med. 2008;46. 10.1016/j.ypmed.2007.11.005 18096216

[82] Riva M, Gauvin L, Richard L. Use of local area facilities for involvement in physical activity in Canada: insights for developing environmental and policy interventions. Health Promot Int. 2007;22:227–35. 17573410 10.1093/heapro/dam015

[83] Pascual C, Regidor E, Martínez D, Elisa Calle M, Domínguez V. Socioeconomic environment, availability of sports facilities, and jogging, swimming and gym use. Health Place. 2009;15:553–61. 18986825 10.1016/j.healthplace.2008.08.007

[84] Karusisi N, Thomas F, Meline J, Chaix B. Spatial accessibility to specific sport facilities and corresponding sport practice: the RECORD Study. Int J Behav Nutr Phys Act. 2013;10:48. 23601332 10.1186/1479-5868-10-48 PMC3641972

[85] Lee RE, Cubbin C, Winkleby M. Contribution of neighbourhood socioeconomic status and physical activity resources to physical activity among women. J Epidemiol Community Health. 2007;61:882–90. 17873224 10.1136/jech.2006.054098 PMC2652966

[86] Liu Y-D, Taylor P, Shibli S. Sport Equity: Benchmarking the performance of English Public Sport facilities. Eur Sport Manag Q. 2009;9:3–21. 10.1080/16184740802461686

[87] Ellaway A, Lamb KE, Ferguson NS, Ogilvie D. Associations between access to recreational physical activity facilities and body mass index in Scottish adults. BMC Public Health. 2016;16. 10.1186/s12889-016-3444-8 PMC4979148 27506767

[88] Bergmann GG, Streb AR, Ferrari M, Alves DCC, Soares BAC, Ferreira GD, et al. The use of outdoor gyms is associated with women and low-income people: a cross-sectional study. Public Health. 2021;190:16–22. 33326889 10.1016/j.puhe.2020.10.024

[89] Gardam KJ, Møller H, Pearson ES. Older adults and outdoor physical activity equipment: a social ecological analysis. Qual Rep. 2021;26:2347–60.

[90] Rovniak LS, Sallis JF, Saelens BE, Frank LD, Marshall SJ, Norman GJ, et al. Adults’ physical activity patterns across Life Domains: Cluster Analysis with replication. Health Psychol. 2010;29:496–505. 20836604 10.1037/a0020428 PMC3021982

[91] Werneck AO, Oyeyemi AL, Araújo RHO, Barboza LL, Szwarcwald CL, Silva DR. Association of public physical activity facilities and participation in community programs with leisure-time physical activity: does the association differ according to educational level and income? BMC Public Health. 2022;22. 10.1186/s12889-022-12593-3 PMC8832843 35148696

[92] Langøien LJ, Terragni L, Rugseth G, Nicolaou M, Holdsworth M, Stronks K et al. Systematic mapping review of the factors influencing physical activity and sedentary behaviour in ethnic minority groups in Europe: a DEDIPAC study. Int J Behav Nutr Phys Act. 2017;14. 10.1186/s12966-017-0554-3 PMC5525226 28738832

[93] Higgerson J, Halliday E, Ortiz-Nunez A, Brown R, Barr B. Impact of free access to leisure facilities and community outreach on inequalities in physical activity: a quasi-experimental study. J Epidemiol Community Health. 2018;72:252–8. 29330166 10.1136/jech-2017-209882 PMC5868528

