# Supplementary material for: The interplay between social environment and opportunities for physical activity within the built environment: a scoping review
Source: BMC Public Health. 2024 Aug 30;24:2361. doi: 10.1186/s12889-024-19733-x (PMC11363614; doi:10.1186/s12889-024-19733-x)
Supplement: Supplementary file 1 — Supplementary Material 1 [file 12889_2024_19733_MOESM1_ESM.docx]

**Appendix 1: Full list of search terms**

| **Search blocks**  **Data-base** | **Physical activity** | **Built environment** | **Social background** |
| --- | --- | --- | --- |
| **Scopus** | Free text words:  Sport* W/2 participati*  Physical W/2 exercis*  Physical W/2 activit*  Active W/2 living  Active W/2 transportati*  Physical W/2 inactivity Sedentary W/2 behavio?r*  Sedentary W/2 lifestyle*  Active W/2 lifestyle*  Recreational W/2 sport* | sport* W/2 facilit*  sport* W/2 hall*  sport* W/2 ground*  sport* W/2 arena*  gym OR gyms  fitness W/2 center*  fitness W/2 centre*  swimming W/2 pool*  leisure W/2 centre*  leisure W/2 center*  recreational W/2 area*  green W/2 space*  sport* W/2 infrastructure*  spatial W/2 accessibilit*  recreational W/2 park*  built W/2 environment  physical W/2 environment  recreational W/2 facility*  neighbo?rhood* W/2 open W/2 space*  bicycl* W/2 path*  walk* W/2 path*  bicycl* W/2 trail*  walk* W/2 trail*  bicycl* W/2 lane*  pedestrian* W/2 facilit*  bicycl* W/2 facilit*  side W/2 walk*  sport* W/2 club*  bicycl* W/2 track* | Free text words:  Socio W/2 economic W/2 status  Socioeconomic W/2 status  social W/2 status  Social W/2 class*  vulnerable W/2 group*  education* W/2 level*  education* W/2 background  education W/2 length  ethnic W/2 minorit*  handicap*  disabilit*  disabled |
| **Sport**  **Discus** | Defined keywords:  DE “SPORTS participation”  D.E. "SEDENTARY behavior"  D.E. "SEDENTARY lifestyles"  D.E. "SEDENTARY people  Free text words:  Sport* N2 participati*  Physical N2 exercis*  Physical N2 activit*  Active N2 living  Active N2 transportati*  Physical N2 inactivity  Sedentary N2 behavio?r*  Sedentary N2 lifestyle*  Active N2 lifestyle*  Recreational N2 sport* | Defined keywords:  D.E. "SWIMMING pools"  D.E. "ACCESSIBLE design of parks"  D.E. "ACCESSIBLE design of playgrounds"  D.E. "ARCHITECTURE & recreation"  D.E. "BICYCLE facilities"  D.E. "TRAILS"  Free text words:  Sport* N2 facilit*  Sport* N2 hall*  Sport* N2 ground*  sport* N2 arena*  gym OR gyms  (Fitness N2 center*) OR (fitness N2 centre*) Swimming N2 pool*  (Leisure N2 centre*) OR (leisure N2 center*) Recreational N2 area*  Green N2 space*  Sport* N2 infrastructure*  Spatial N2 accessibilit*  Recreational N2 park*  Built N2 environment  Physical N2 environment  Recreational N2 facility*  Neighbo?rhood* N2 open N2 space*  Bicycl* N2 path*  Walk* N2 path*  Bicycl* N2 trail*  Walk* N2 trail*  Bicycl* N2 lane*  Pedestrian* N2 facilit*  Bicycl* N2 facilit*  Side N2 walk*  Sport* N2 club*  Bicycl* N2 track* | Defined keywords:  D.E. "MINORITIES in sports"  D.E. "PEOPLE with disabilities"  Free text words:  Socio N2 economic N2 status  socioeconomic N2 status  Social N2 class*  Vulnerable N2 group*  Education* N2 level*  Education* N2 background  Education N2 length  Social N2 status  Ethnic N2 minorit*  Handicap*  Disabilit* OR Disabled |
| **Global Health** | Defined keywords:  D.E. "sport"  D.E. "physical activity"  D.E. "active recreation"  Free text words:  Same as SPORTdiscus | Defined keywords:  D.E. "sports facilities"  D.E. "sports centres"  D.E. "sports grounds"  D.E. "leisure centres"  D.E. "recreational facilities"  Free text words:  Same as SPORTdiscus | Defined keywords:  D.E. "socioeconomic status"  D.E. "social classes"  D.E. "social status"  D.E. "ethnic groups"  D.E. "people with disabilities"  Free text words:  Same as SPORTdiscus |
| **Socio-**  **logical** | Defined keywords:  MAINSUBJECT.EXACT("Sports Participation")  Free text words:  Sport* NEAR/2 participati* Physical NEAR/2 exercis* Active NEAR/2 living  Active NEAR/2 transportati*  Physical NEAR/2 inactivity  Sport* NEAR/2 participati*  Physical NEAR/2 exercis* Physical NEAR/2 activit* Active NEAR/2 living  Active NEAR/2 transportati*  Physical NEAR/2 inactivity  Sedentary NEAR/2 behavio?r*  Sedentary NEAR/2 lifestyle*  Active NEAR/2 lifestyle*  Recreational NEAR/2 sport* | Defined keywords:  MAINSUBJECT.EXACT("Built Environment")  MAINSUBJECT.EXACT.EXPLODE("Recreational Facilities")  Free text words:  Sport* NEAR/2 facilit*  Sport* NEAR/2 hall*  Sport* NEAR/2 ground*  sport* NEAR/2 arena*  gym OR gyms  Fitness NEAR/2 center*  fitness NEAR/2 centre*  Swimming NEAR/2 pool*  Leisure NEAR/2 centre*  leisure NEAR/2 center*  Recreational NEAR/2 area*  Green NEAR/2 space*  Sport* NEAR/2 infrastructure*  Spatial NEAR/2 accessibilit*  Recreational NEAR/2 park*  Built NEAR/2 environment  Physical NEAR/2 environment  Recreational NEAR/2 facility*  Neighbo?rhood* NEAR/2 open NEAR/2 space*  Bicycl* NEAR/2 path*  Walk* NEAR/2 path*  Bicycl* NEAR/2 trail*  Walk* NEAR/2 trail*  Bicycl* NEAR/2 lane*  Pedestrian* NEAR/2 facilit*  Bicycl* NEAR/2 facilit*  Side NEAR/2 walk*  Sport* NEAR/2 club*  Bicycl* NEAR/2 track* | Defined keywords:  MAINSUBJECT.EXACT.EXPLODE("Socioeconomic Status")  MAINSUBJECT.EXACT.EXPLODE("Social Class")  MAINSUBJECT.EXACT.EXPLODE("Social Status")  MAINSUBJECT.EXACT.EXPLODE("Handicapped")  Free text words:  Socio NEAR/2 economic NEAR/2 status  socioeconomic NEAR/2 status  Social NEAR/2 class*  Vulnerable NEAR/2 group*  Education* NEAR/2 level*  Education* NEAR/2 background  Education NEAR/2 length  Social NEAR/2 status  Ethnic NEAR/2 minorit*  Handicap*  Disabilit* OR Disabled |
