# Supplementary material for: The interplay between social environment and opportunities for physical activity within the built environment: a scoping review
Source: BMC Public Health. 2024 Aug 30;24:2361. doi: 10.1186/s12889-024-19733-x (PMC11363614; doi:10.1186/s12889-024-19733-x)
Supplement: Supplementary file 2 — Supplementary Material 2 [file 12889_2024_19733_MOESM2_ESM.docx]

**Appendix 2: Spreadsheet of the 72 included articles in alphabetical order (numbering corresponds with reference number)**

| **#** | **Author(s), year, title** | **Country(-ies)** | **Study design and methods** | **Population(s) and/or built environment(s)** | **Aim and variable categories** | **Main results and conclusions** | **Type of Built Environment** |
| --- | --- | --- | --- | --- | --- | --- | --- |
| 49 | Adkins, A., Makarewicz, C., Scanze, M., et al. (2017). *Contextualizing Walkability: Do Relationships Between Built Environments and Walking Vary by Socioeconomic Context?* | USA, Canada, Australia, Sweden, and Belgium | Literature review (n=17) | Disadvantaged adults above 18 years, two studies on elderly and one on young persons. | What influence do socio-economic conditions (income, ethnicity, education) have on connections between walking and the built environment (walkability)? | Across the 17 studies, this review finds that the environment is of less importance for walking for groups with lower income or less education. There is a tendency for this group to walk relatively more in areas with lower walkability, and conversely less in areas with higher walkability. The difference is explained by several factors. | Walkability |
| 48 | Andrade, L., Geffin, R., Maguire, M., et al. (2021) *The Associations Between Access to Recreational Facilities and Adherence to the American Heart Association’s Physical Activity Guidelines in US Adults.* | USA | Cross sectional study, using survey data (N=1,750) | Adults above 18 years | We investigated whether access to free or low-cost recreational facilities was associated with meeting the American Health Association PA guidelines. Covariates included age, sex, level of education, overall health, BMI, ethnicity, hours of work per week, income, and time living at current address. | The study suggests that improving access to affordable recreational facilities (parks, walking trails, bike paths and courts) in neighborhoods could promote physical activity and potentially lead to better health outcomes, especially for those with lower access to such facilities and lower income levels. However, the study also highlights disparities in access based on income and other factors that should be considered when developing strategies to promote physical activity and health in communities. | Walkability and Cyclist infrastructure |
| 69 | Anthun, K.S., Maass, R.E.K., Hope, S., et al. (2019). *Addressing Inequity: Evaluation of an Intervention to Improve Accessibility and Quality of a Green Space.* | Norway | Mixed-method research design consisting of counting data, surveys, registry data and interviews | Adults above 18 years (users of the green space area) | The use of a green space area and whether and how the area was beneficial for health, social inclusion, and physical activity for all socioeconomic groups in a suburban area in Norway | The study showed significantly increased use of the area from 2015–2018 and that users belonged to different socioeconomic groups. The motivation for using the area was the opportunity to experience nature and to interact socially. While no significant changes in self-rated health, life satisfaction, or levels of physical activity were found, the study indicates that factors such as location, availability, and designated places for social interaction are important motivating factors for use. Users from the lower socioeconomic groups were among the frequent users but were also the least satisfied with the quality and availability of the path. However, the relationship between income and perceptions of the path are complex. The only personal variable that remained significant throughout the last step of the analysis was income, which displayed a negative relationship with use of the path: the less household income participants had, the more likely they were to use the path very often. | Neighborhood parks and open spaces |
| 88 | Bergmann, G.G., Streb, A.R., Ferrari, M., et al. (2021). *The use of outdoor gyms is associated with women and low-income people: A cross-sectional study.* | Brazil | Population-based cross sectional study using interview data (n=431) | Adults above 18 years | To examine the prevalence, and the demographic, socio-economic, and health correlates to Outdoor Gyms (OGs) use for adults from a southern Brazilian city. | About 30% of the population uses outdoor gyms for PA practice. Women and low-income people are those who more commonly use outdoor gyms for PA practice. | Sports facilities |
| 46 | Besor, O., Paltiel, O., Manor, O., et al. (2021). *Associations between density and quality of health promotion programmes and built environment features across Jerusalem.* | Jerusalem | Cross sectional study, using GIS and survey data (n=1685) | Adults between 18-75 years | This study aims to examine the associations between the distribution and quality of health promoting programs in the metropolis of Jerusalem, along with health characteristics, area-level SES and urban characteristics enabling PA. | Areas with more walking paths and areas characterized by residents from lower SES had better performing Health promotion program. Higher European quality instrument for health promotion scores were also found in areas where a higher proportion of the Arab minority population resides. | Walkability and Cyclist infrastructure |
| 75 | Billaudeau, N., Oppert, J.M., Simon, C., et al. (2011). *Investigating disparities in spatial accessibility to and characteristics of sport facilities: Direction, strength, and spatial scale of associations with area income.* | France | Spatial analysis of the distribution of sport facilities | Sport facilities (area level) | To look at the distribution of sports facilities and their accessibility in Paris and the importance of the areas SES and quality of facilities | The study finds no correlation between the socio-economic status of local areas and a lower availability of sports facilities or that the quality of the facilities is worse in these areas. However, the study finds that certain facility types are less accessible in local areas with low SES. This concerns, among other things, tennis courts and fitness centers. On the other hand, facility types such as ball fields and larger common areas are more available here than in areas with high SES. It is discussed whether the distribution of certain types of facilities is related to the prices of building plots and the preferred activities of the population in the respective areas (high/low SES). | Sports facilities |
| 40 | Boone-Heinonen, J. & P. Gordon-Larsen (2011). *Life stage and sex specificity in relationships between the built and socioeconomic environments and physical activity.* | USA | Cross sectional study, using data from the National Longitudinal Study of Adolescent Health (n =12,701) and linked geographical information. | Young males and females between 11-22 years | The study examines the cross-sectional associations between several built and socioeconomic environment characteristics and physical activity (MVPA) among males and females in different life stages. | Higher landscape diversity and lower crime were related to greater weekly MVPA regardless of sex or life stage. Higher Street connectivity was marginally related to lower MVPA in females but not males. Pay facilities and public facilities per 10,000 population and median household income were unrelated to MVPA | Walkability and Sports facilities |
| 35 | Buli, B.G., Tillander, A., Fell, T., et al. (2022). *Active Commuting and Healthy Behavior among Adolescents in Neighborhoods with Varying Socioeconomic Status: The NESLA Study.* | Sweden | Cross sectional study, using survey data (n=314) | Young people between 16-19 years | This study aimed to assess factors associated with modes of transportation to and from school among adolescents aged 16–19 living in a middle-sized city in Sweden. Focusing on active commuting determined by various factors, including the socioeconomic status (SES) of families and neighborhoods, distance to schools, perceived neighborhood safety, lifestyles and availability of walkways and biking paths. | Adolescents living in high SES neighborhoods were 80% more likely to bike or walk to school than adolescents living in low SES neighborhoods. Furthermore, active commuting was 50% less common among adolescents from neighborhoods with middle SES than among those in low SES neighborhoods. In contrast, subjectively reported individual family economic status was not associated with active commuting to and from school. | Walkability and Cyclist infrastructure |
| 57 | Burton, N.W., Turrell, G., & Oldenburg, B. (2003). *Participation in recreational physical activity: Why do socioeconomic groups differ?* | Australia | Qualitative interview study (n=60) | Adults between 18-60 years, 10 women and 10 men with different income levels | This study explored how influences on recreational physical activity (RPA) were patterned by socioeconomic position. | Influences salient across all groups included previous opportunities, physical health, social assistance, safety, environmental aesthetics and urban design, physical and health benefits, and barriers of self-consciousness, low skill, and weather/time of year. Influences more salient to the high socioeconomic group included social benefits, achieving a balanced lifestyle, and the barrier of an unpredictable lifestyle. Influences more salient to the high and mid socioeconomic groups included efficacy, perceived need, activity demands, affiliation, emotional benefits, and the barrier of competing demands. Influences more salient to the low socioeconomic group included poor health and barriers of inconvenient access and low personal functioning. | Walkability and Sports facilities |
| 76 | Cereijo, L., Gullón, P., Cebrecos, A., et al. (2019). *Access to and availability of exercise facilities in Madrid: an equity perspective.* | Spain | Cross sectional study, using geocoding, registry data and counting data. | Sport facilities (area level) | This study analyzed the relationship between area-level socioeconomic status (SES) and access to, and availability of, exercise facilities in Madrid, Spain. | Lower SES areas had a lower average distance to the closest facility, especially for public and low-cost facilities. Higher SES areas had higher availability of exercise facilities, especially for private and seasonal facilities. | Sports facilities |
| 33 | Cerin, E. & E. Leslie (2008). *How socio-economic status contributes to participation in leisure-time physical activity.* | Australia | Cross sectional study, using survey data (n=2.194) and population data. | Adults between 20-65 years | The aim of this study was to identify individual, social, and environmental contributors (mediators) to individual- and area-level differences in leisure-time physical activity across socio-economic groups. | Respondents with lower income indicated poorer access to facilities and more physical barriers to walking in the immediate area, while respondents who lived in areas with lower income had more open spaces, more team sports facilities, but less aesthetically pleasing surroundings and fewer individual sports facilities. The mediation analyzes of the relationship between SES and physical activity showed that the personal and social factors explained strong parts. Efforts in areas with lower SES must therefore use a holistic approach and promote social and individual factors at the same time as improvements to the built environment. | Walkability and Sports facilities |
| 32 | Choi, Y. & Yoon, H. (2020) *Do the Walkability and Urban Leisure Amenities of Neighborhoods Affect the Body Mass Index of Individuals? Based on a Case Study in Seoul, South Korea.* | South Corea | Cross sectional study, using survey data (n=577) and geographic information data (SGIS). | Adults between 19-64 years | Does the neighborhood environment, along with socioeconomic characteristics (income and education), impact individuals’ degree of obesity, controlling for dietary pattern and physical activity? | The SES is negatively associated with walkability, meaning that, on average the people of higher SES live in less walkable neighborhoods. On the other hand, the SES is positively associated with urban leisure amenities, revealing that more urban leisure amenities are supplied in wealthier neighborhoods than elsewhere. | Walkability and Sports facilities |
| 39 | Christie, C.D., Consoli, A., Ronksley, P.E. et al. (2021) Associations between the built environment and physical activity among adults with low socio-economic status in Canada: a systematic review. | Canada | Systematic review (PROSPERO) | Adults with low SES above 18 years and living in Canada | To synthesize literature on the associations between the built environment and physical activity among adults with low socio-economic status (SES) in Canada. | Findings suggest that the neighborhood-built environment is associated with physical activity among adults with low SES in Canada. More rigorous study designs are needed to determine whether the built environment and physical activity are causally related within this vulnerable population. | Walkability |
| 47 | Christie, C.D., Friedenreich, C.M., Vena, J.E. et al. (2022). *Cross-sectional and longitudinal associations between the built environment and walking: effect modification by socioeconomic status.* | Canada | Cross-sectional study, using survey data (n=703) | Adults between 35-69 years | This study examines 1) whether overall neighborhood walkability and specific built characteristics were associated with walking among adults at a single point in time and after they relocate neighborhoods, and 2) test for effect modification of these associations by SES. | The study found negative cross-sectional associations between street connectivity and walking among adults with lower education and income, and a positive association between percent change in walkability and change in walking among lower educated adults. Further, that changes in neighborhood walkability, resulting from residential relocation, might more strongly affect walking among low SES adults. | Walkability |
| 27 | Clary, C., Lewis, D., Limb, E., Nightingale, C., et al. (2020a) *Longitudinal impact of changes in the residential built environment on physical activity: findings from the ENABLE London cohort study.* | England | Co-hort study, using accelerometer data and GIS-derived measures (n=687) - Part of ENABLE London) | Adults above 16 years | This paper examines whether changes in a range of residential built environment features are associated with changes in measures of physical activity in adults. It also explores whether observed effects are moderated by socio-economic status, measured by housing tenure (social, intermediate, market-rent). | The study did not find evidence that increased accessibility to greenspace was associated with change in PA level at follow-up. This finding held true for the three housing groups. On the contrary, they found that there was a correlation with increased access to public transport, where housing seekers decreased their physical activity level with increased access, and market rent house seekers increased their physical activity level. | Walkability and Neighborhood parks and open spaces |
| 28 | Clary, C., Lewis, D., Limb, E.S., et al. (2020b). *Weekend and weekday associations between the residential built environment and physical activity: Findings from the ENABLE London study.* | England | Study using baseline measurements of PA and sociodemographic data from the ENABLE London study. Consist of accelerometer data and GIS-derived measures (n = 1,064) | Adults above 16 years seeking “social”, “intermediate” and “market-rent” accommodation in East Village. | This study assessed whether the residential built environment was associated with physical activity (PA) differently on weekdays and weekends, and contributed to socio-economic differences in PA. | The residential built environment is associated with PA differently at weekends and on weekdays, and contributes moderately to socio-economic differences in PA. | Walkability and Neighborhood parks and open spaces |
| 58 | Cleland, V.J., Ball, K., Salmon, J., et al. (2010). *Personal, social, and environmental correlates of resilience to physical inactivity among women from socio-economically disadvantaged backgrounds* | Australia | Cross sectional study, using survey data (n=291) | Women above 18 years of low socio-economic position | This study examines characteristics of women from low-income conditions who, despite their situation, are physically active. The characteristics are divided into the personal-, social- and environmental factors for physical activity. | The strongest explanatory factors for PA must be found at the personal level: the enjoyment of the activity and self-efficacy, where the social factors and environmental factors are fewer explanatory factors for why women with a low income are physically active. | Walkability |
| 65 | Cohen, D.A., Han, B., Park, S., et al. (2022). *Park Use and Park-Based Physical Activity in Low-Income Neighborhoods.* | USA | Cross sectional study, using survey data (2.973) | Park users in low-income areas, above 18 years. | This study examines factors associated with park use among a representative sample of adult residents of low-income neighborhoods who live within a 1-mile radius of neighborhood parks. | The study found that women’s visits to parks in low-income areas generally is centered around children, whereas men’s visits were more likely to be associated with their own physical activity. Barriers for seniors are associated with limited facilities and programming that meet their needs. Individuals with part time job, visits parks more often than unemployed. The number of park visits and duration of stay were not associated with the rate of violent crime but were positively associated with individual perception of safety. Further, that the closer you live to the park, the more often you visit the park, Latinos visit the park less than white/others, part-time people visit the park more than persons without work, and college educated also visit green parks more often than high school educated. | Neighborhood parks and open spaces |
| 50 | Compernolle, S., De Cocker, K., Roda, C., et al. (2016). *Physical environmental correlates of domain-Specific Sedentary behaviours across five European regions (the SPOTLIGHT Project).* | France, United Kingdom, Belgium, and the Netherlands | Cross sectional study, using surveys and (n=5.205) objective analysis from google street view. | Adults above 18 years | This study firstly aims to examine the association of perceived and objectively measured neighborhood safety, aesthetics, destinations, and functionality with transport-related, work-related and leisure-time sedentary behavior. Secondly, the study aims to assess whether these associations are moderated by age, gender, or educational level. | Lower levels of leisure-time sedentary behavior were observed among adults who perceived greater numbers of destinations such as supermarkets, recreational facilities, or restaurants in their neighborhood, and among adults who lived in a neighborhood with more objectively measured aesthetic features, such as trees, water areas or public parks. Lower levels of work-related sedentary behavior were observed among adults who perceived fewer aesthetic features in their neighborhood, and among adults who lived in a neighborhood with less objectively measured destinations. Both age, gender and educational level moderated the associations between neighborhood environmental factors and sedentary behaviors. | All |
| 2 | Conderino, S.E., Feldman, J.M., Spoer, B., et al. (2021). *Social and Economic Differences in Neighborhood Walkability Across 500 U.S. Cities.* | USA | Cross sectional study, using measures for walk score across 500 cities in USA. | 497 of the most populous cities in the U.S. and the 3 highest population cities in Vermont, West Virginia, and Wyoming as of 2010 to allow for representation from all 50 states | This study examines univariable and multivariable associations between sociodemographic factors and walkability across 500 major urban centers in the U.S., allowing for consistent inferences that are representative of all large cities across the nation. | The study found a relationship between income level and walkability, with low-income neighborhoods associated with higher average Walk Scores than high-income neighborhoods. Overall, majority White neighborhoods had, on average, lower Walk Scores than other racial/ethnic majority neighborhoods or neighborhoods with no racial/ethnic majority. However, this association was reversed within majority Black neighborhoods, where tracts in lower income tertiles had the lowest walkability. | Walkability |
| 8 | Cutumisu, N. & Spence, J.C. (2012). *Sport fields as potential catalysts for physical activity in the neighbourhood.* | Canada | Cross sectional study, using survey data (n=2,879), Neighborhood-level data and GIS data. | Adults above 18 years | This study investigates the association between the objective and perceived accessibility of sport fields and the levels of self-reported physical activity among adults in Edmonton, Canada. | The study found that access to facilities (objectively measured) together with personal factors such as age, gender, and level of education as well as self-efficacy increases the likelihood that individuals will undertake the recommended levels of physical activity. However, there were no correlations between individuals' subjective perception of access to facilities and their participation in PA. There is thus a difference between objective and subjective assessment of the surroundings. | Sports facilities |
| 36 | Darcy, M., Parkinson, J., McDonald, N., et al. (2022). *Geographic remoteness and socioeconomic disadvantage reduce the supportiveness of food and physical activity environments in Australia.* | Australia | An observational, cross-sectional study. | Twenty-five communities in Australia. | The aim of this study was to comprehensively assess the community food and physical activity environments from a range of communities spread across Queensland, particularly those of socially disadvantaged populations. | The function of the physical activity environment and remoteness area negatively correlated. This indicates that more remote areas had less functional physical activity environments, that is, they have poorer structural aspects relating to the physical attributes and quality of the streets and pathways. And more disadvantaged areas, often residential areas, within the same local government area have access to lower quality physical activity environments. Further, living in more disadvantaged areas generally have less opportunity to eat healthy and to undertake physical activity in a safe and accessible environment. | Walkability and Cyclist infrastructure |
| 56 | Dias, A.F., Gaya, A.R., Santos, M.P., et al. (2020). *Neighborhood environmental factors associated with leisure walking in adolescents.* | Brazil | Cross sectional study, using survey data (n=1,113), WALK-scores and GIS data. | Teenagers between 14-20 years | The study aims to verify the associations of leisure walking with perceived and objective measures of neighborhood environmental factors stratified by gender and socioeconomic status (SES) in Brazilian adolescents. | Leisure walking was positively associated with access to services and lower distance to parks and squares in girls from low SES. Residential density and walkability index were associated with leisure walking in girls from middle SES. Boys from low SES showed an inverse association between crime safety and leisure walking. Neighborhood recreation facilities was positively associated with leisure walking in middle SES. Land use mix, neighborhood recreation facilities and places for walking were positively associated with leisure walking in high SES. | Walkability, Cyclist infrastructure |
| 26 | Doiron, D., Setton, E.M., Shairsingh, K., et al. (2020). *Healthy built environment: Spatial patterns and relationships of multiple exposures and deprivation in Toronto, Montreal and Vancouver.* | Canada | The study is using nationally standardized estimates of active living friendliness (i.e. “walkability”), NO2 air pollution, and greenness within four cities based on deprivation scores. All environmental exposures and deprivation scores were provided by CANUE. | Three large cities in Canada that provide a representative picture of the different areas in relation to low/high SES in the local areas. | The study aims to explore the spatial patterns of urban environmental exposures within three large Canadian cities, assess how exposures are distributed across socio-economic deprivation gradients and identify clusters of favorable or unfavorable environmental characteristics. | In all three cities, high walkability was more common in least deprived areas and less prevalent in highly deprived areas. We also generally saw a greater prevalence of postal codes with relatively high greenness indices and low NO2 air pollution in areas with low deprivation, and a lower greenness prevalence and higher NO2 concentrations in highly deprived areas, suggesting environmental inequity is occurring. The most materially deprived areas of Toronto, Montreal and Vancouver were around half as likely to be highly walkable, whereas high walkability was between 68% and 114% more prevalent in the least deprived postal codes. Persons living in Toronto, Montreal, and Vancouver neighborhoods of high deprivation were also about half as likely to be surrounded by high greenness, relative to city-wide high greenness prevalence. | Walkability and Neighborhood parks and open spaces |
| 70 | Eime, R.M., Harvey, J., Charity, M.J., et al. (2017). *The relationship of sport participation to provision of sports facilities and socioeconomic status: a geographical analysis* | Australia | Cross sectional study, using membership registration data (n=488.693) and GIS data. | Children between 5-14 years and young people and adults above 15 years being active in specific activities | This study examined the geographical association between provision of sport facilities and participation in sport across an entire Australian state, using objective total enumerations of both, for a group of sports, with adjustment for the effect of socioeconomic status (SES). | The study finds that there is great variation in sports participation and facility density within the state, which is attributed to differences in population and facility density (and availability). There is a positive correlation between participation in specific activities and the presence of facilities for the activities in question, regardless of whether it is in urban areas or in rural areas. These relationships still exist when controlling for SES. Rural areas have better participation and better facility coverage than urban areas. However, the study cannot say in which direction the causality goes. | Sports facilities |
| 87 | Ellaway, A., Lamb, K.E., Ferguson, N.S., et al. (2016). *Associations between access to recreational physical activity facilities and body mass index in Scottish adults.* | Scotland | Cross sectional study, using national survey data (n=6,365) and GIS-data. | Adults above 18 years | The aim of this study was to link individual health and behavioral data with area-level spatial data to examine whether the body mass index (BMI) of adults was associated with access to recreational physical activity (PA) facilities by different modes of transport (bus, car, walking, cycling) and the extent to which any associations were mediated by PA participation. | The study finds correlations between accessibility to sports facilities and low BMI. However, no significant correlation is found between accessibility and physical activity when controlling for age, gender, SES, illness, dietary habits, access to a car and urbanization. | Sports facilities |
| 73 | Farrell L., Hollingsworth B., Propper C., Shields M.A. (2014) *The socioeconomic gradient in physical inactivity: Evidence from one million adults in England* | England | Cross sectional survey from Active People Survey 2004-2011 | 1,002,216 adults aged 16 and over from various regions in England. | To understand the association between socioeconomic status and physical inactivity. Variable Categories: Physical inactivity, education level, household income, area deprivation, availability of physical recreation/sporting facilities, local weather, regional geography. | The study found high levels of physical inactivity in the population. Both education and household income were strongly associated with physical inactivity. The gap in inactivity between high and low-income households was evident from early adulthood and widened up to about age 85. The findings suggest significant future health problems in England, with a heavy social gradient. | Sports facilities |
| 79 | Ferguson, N.S., Lamb, K.E., Wang, Y., et al. (2013). *Access to Recreational Physical Activities by Car and Bus: An Assessment of Socio-Spatial Inequalities in Mainland Scotland.* | Scotland | GIS analysis and area specific register data. | Analysis at area level, where 6,412 data zones were analyzed. | This paper explores the distribution of access to PA facilities by car and bus across mainland Scotland by income deprivation at data zone level. | Access to PA facilities by car was significantly (p,0.01) higher for the most affluent quintile of area-based income deprivation than for most other quintiles in small towns and all other quintiles in rural areas. Accessibility by bus was significantly lower for the most affluent quintile than for other quintiles in urban areas and small towns, but not in rural areas. Overall, we found that the most disadvantaged groups were those without access to a car and living in the most affluent areas or in rural areas. | Sports facilities |
| 63 | Fontán-Vela, M., Rivera-Navarro, J., Gullón, P., et al. (2021). *Active use and perceptions of parks as urban assets for physical activity: A mixed-methods study.* | Spain | Convergent-parallel mixed-methods study, using observations, semi-structured interviews (n=37) and focus groups interviews (n=29). | Adult (above 40 years) users of the park in the six selected parks, which was selected due to their NSES-level. | Analyze differences in park use and physical activity (PA), and the perceptions of parks as urban assets for PA. | Parks within the high-Neighborhood SES (NSES) were more visited, showing a higher proportion of people performing high PA as compared to residents of the middle and low-NSES. Female visitors showed lower PA levels compared to men, especially for parks within high-NSES. Residents from high-NSES reported fewer barriers to park use compared to residents from the other areas, who reported limitations such as less leisure time due to job constrains or perceived insecurity in parks. Although there were public parks in the low-SES neighborhood, the above-mentioned factors caused many neighbors to perceive that they could not use them and to use other parks located in other neighborhoods. Senior participants reported that having parks with organized activities and a design oriented towards different age-groups are valuable. | Neighborhood parks and open spaces |
| 43 | Frost, S.S., Goins, R.T., Hunter, R.H., et al. (2010). *Effects of the built environment on physical activity of adults living in rural settings.* | USA, Canada, and Australia | Systematic review including 16 quantitative studies and 4 qualitative studies. | Adults above 18 years | To conduct a systematic review of the literature to examine the influence of the built environment (BE) on the physical activity (PA) of adults in rural settings. | There was a positive correlation between physical activity and the built environment on the following parameters: the perception of aesthetics-, paths, safety against crime or traffic-, the presence of recreational facilities-, parks and the ease of walking between destinations in the environment for adults in rural districts. Sidewalks, traffic, and street lighting were three parameters that were not consistently associated with a positive association with physical activity for adults in rural areas. | Walkability, Neighborhood parks and open spaces and Sport facilities |
| 66 | García-Pérez, H., & Lara-Valencia, F. (2021). *Association between neighborhood parks and leisure-time physical activity among adult mexican women.* | Mexico | Cross sectional study, using survey data (n=1,285) | Adult women between 25-54 years in the city Hermosillo | This study examines whether women’s leisure-time physical activity (LTPA) is associated with neighborhood socioeconomic status, presence of public parks, and sociodemographic and health characteristics of women living in a mid-size Mexican city | Our results indicate no evidence that the number of parks, park-to-people ratio, frequency of combined service areas, or distance to the nearest park have any influence on physical activity among women aged 25 to 54 years old in Hermosillo. On one hand, women residing between 350 to 750 meters from a park had an increase of 48% in the odds of walking and running relative to women living at more distant locations. On the other hand, SES increased the odds of women’s engagement in neighborhood-based physical activity by 8% after controlling for park related variables. After controlling for neighborhood-level variables, women’s age and education were also statistically associated with physical activity. | Neighborhood parks and open spaces |
| 89 | Gardam, K. J., Møller, H., & Pearson, E.S. (2021). *Older Adults and Outdoor Physical Activity Equipment: A Social Ecological Analysis.* | Canada | Case study design, using a socioecological model (SEM), qualitative interviews, observations, and document reviews. | Older adults for interviews, but for observations everyone was included, which was mostly people 18 years and younger, and the rest was between 19-54, and only two above 54 years. | The purpose of this study was to explore and identify the social ecological factors that influenced older adults’ uptake of an Outdoor adult playground (OAP) installed in a neighborhood of low-socioeconomic status. | The interaction between individuals of all ages and the social and built environments can have a significant impact on the uptake of equipment use. Difficulties understanding the instructional plaques negatively impacted those who perhaps had not used exercise equipment previously. Individual and neighborhood socioeconomic status combined with the location of the OAP produced a situation where inequities in access to physical activity infrastructure may have been reduced. | Sports facilities |
| 61 | Garrett, J.K., White, M.P., Elliott, L.R., et al. (2020). *Urban nature and physical activity: Investigating associations using self-reported and accelerometer data and the role of household income.* | England | Cross-sectional study, using survey and accelerometer data. | Adults above 16 years | The study explored the associations between meeting physical activity (PA) guidelines and both neighborhood green (area coverage) and blue (freshwater coverage and coastal proximity) environments for urban adults. Relationships were stratified by equivalized household income as an indicator of socio-economic status. | The study shows that there is a difference in one's physical activity between groups and different incomes in relation to how much green areas and freshwater constitute the area in question. For example, those with the lowest income who live in the greenest areas (80-100%) are most likely to meet the guidelines for physical activity in recreational contexts, rather than in sports clubs and by walking. There is therefore a connection between the place you live and the surroundings in relation to which type of physical activity you use the most, and this varies further across income. Immediately, the opportunities are more important for the people with the lowest income. | Neighborhood parks and open spaces |
| 51 | Giles, L.V. (2021). *When physical activity meets the physical environment: precision health insights from the intersection.* | USA and Australia | Review study (n=14) | N/A | This study investigates four specific aspects of the physical environment, walkability, green space, traffic related air pollution and heat, and how we can enhance our ability to precisely guide physical activity in the context of the spaces within which we live and move. | Strategies to increase physical activity could include optimizing design of the built environment or mitigating of some of the environmental impediments to activity through personalized or population-wide interventions. | Walkability and Neighborhood parks and open spaces |
| 30 | Giles-Corti, B. & Donovan, R.J. (2002). *Socioeconomic status differences in recreational physical activity levels and real and perceived access to a supportive physical environment.* | Australia | Cross sectional study, using survey data (n=1,803) | Adults between 18-59 years | Based on the respondent’s area of residence, this study examines SES differences in patterns of physical activity and access to a supportive physical environment. | Respondents in low SES areas had superior spatial access to many recreational facilities but were less likely to use them compared with those living in high SES areas. They were more likely to perceive that they had access to sidewalks and shops, but also perceived that their neighborhood was busier with traffic, less attractive, and less supportive of walking. After adjustment, respondents living in low SES areas were 36% less likely to undertake vigorous activity. While they were more likely to walk for transport, this was not statistically significant, nor were other SES differences in walking for recreation and walking as recommended. Modifiable environmental factors were associated with walking and vigorous activity, especially perceived access to sidewalks and neighborhood attractiveness. Spatial access to attractive, public open space was associated with walking. | Walkability and Sports facilities |
| 54 | Gullon, P., Bilal, U., Hirsch, J.A., et al. (2020). *Does a physical activity supportive environment ameliorate or exacerbate socioeconomic inequities in incident coronary heart disease?* | USA | Using data from the co-hort study Reasons for Geographic and Racial Differences in Stroke from 2003-2007 (n=20,808) | Adults above 45 years | This study aimed to explore whether socioeconomic inequities in coronary heart disease (CHD) incidence are ameliorated or exacerbated in environments supportive of physical activity (PA) | We found that REGARDS participants with lower socioeconomic position had a higher incidence of CHD. These inequities were relatively wide in areas with no walking destinations. In contrast, the income–CHD association showed a trend towards being strongest in areas with the highest percentage of green land cover. | Walkability, Neighborhood parks and open spaces |
| 93 | Higgerson, J., Halliday, E., Ortiz-Nunez, A., et al. (2018) *Impact of free access to leisure facilities and community outreach on inequalities in physical activity: a quasi-experimental study* | England | Intervention study, using local  administrative data and a large national survey (n=1,556,563) | Adults above 16 years | What change occurs in the use of sports facilities, if access is free and an increased marketing and/invitation effort? And does these changes differ by socioeconomic groups. | The study suggests that removing user charges from leisure facilities in combination with outreach and marketing activities can increase overall population levels of physical activity while reducing inequalities. Showed by a 64% improvement in the use of the facilities could be measured after the effort. The measurement at population level showed an increase of 4% in the proportion of physically active citizens, which was more pronounced for citizens employed in crafts and industry or without a job. | Sports facilities |
| 53 | Hillsdon, M., Coombes, E., Griew, P., et al. (2015). *An assessment of the relevance of the home neighbourhood for understanding environmental influences on physical activity: How far from home do people roam?* | England | Cross sectional, population-based study, using interviews, accelerometer, and GPS data (n=195). | Adults above 18 years | This study aimed to examine the distance from home at which physical activity takes place and how this varies by personal and neighborhood characteristics | The study finds that most people go further than 800 m (10 m walk) from their home/neighborhood to be active, which is why the character of a neighborhood is therefore poor at predicting whether people are active. Men travel further than women to be active, as do rural people, higher SES neighborhoods and car owners. | Walkability |
| 78 | Hillsdon, M., Panter, J., Foster, C., Jones, A. (2007). *Equitable Access to Exercise Facilities* | England | The study used a database of all indoor exercise facilities in England, linked to administrative areas with a deprivation score. The analysis involved calculating the density of physical activity facilities per 1,000 people per quintile of deprivation, using census data. | General population of England, with a focus on different quintiles of deprivation based on administrative areas. | To examine the relationship between neighborhood deprivation and the density of physical activity facilities in England. Variable Categories: Deprivation levels, type, and density of physical activity facilities. | The study found a significant negative relationship between area deprivation and the density of physical activity facilities. The availability of physical activity facilities declines with the level of deprivation, indicating that areas in most need of facilities to support active lifestyles have fewer resources. This suggests the need for a more equitable distribution of opportunities to participate in physical activity. | Sports facilities |
| 71 | Hoekman, R., Breedveld, K., & Kraaykamp, G. (2017). *Sport participation and the social and physical environment: explaining differences between urban and rural areas in the Netherlands.* | The Netherlands | Cross sectional study, using a socio-ecological theoretical model, survey data (n=17,910), social and physical environment variables, and GIS data. | Individuals between 6 and 79 years. | This study investigated the intensity of sport participation in the Netherlands comparing urban and rural areas. | The study does not find a big difference in weekly sports participation between rural and urban areas. What matters more for sports participation is the SES of people's local area and to some extent the number of different types of facilities available. The study emphasizes the need to include all levels (individual, facility, local area, etc.) in explanatory models for sports participation. | Sports facilities |
| 41 | Isiagi, M. Okop, K.J. & Lambert, E.V. (2021). *The Relationship between Physical Activity and the Objectively-Measured Built Environment in Low- and High-Income South African Communities.* | South Africa | Cross sectional study, using survey data (n=52), accelerometer and GIS data. | Adults between 18 and 65 years. | This study explored the extent to which objectively measured attributes of the built environment were associated with self-report or device-measured PA in low- and high-socioeconomic status (SES) communities. | The device-measured physical activity for all groups was inversely associated with intersection density. There were also observed differences in transport-related, self-report physical activity between SES groups. Residents in the low-SES/high walkable neighborhoods reported more transport-related physical activity compared to high-SES/low walkable. There was a significant overall difference in device-measured vigorous physical activity between income groups, with between group differences for the low-SES/low walkable vs. high-SES/low walkable groups. | Walkability |
| 29 | Jacobs, J., Alston, L., Needham, C., et al. (2019). *Variation in the physical activity environment according to area-level socio-economic position— A systematic review.* | USA, Australia, France, Scotland, Germany, Canada, Spain, England, Portugal, New Zealand, and Denmark. | Systematic review, using six different databases (n=59) | N/A | This study aims to identify whether the physical activity environment varies by socio‐economic position (SEP), which may contribute to socio‐economic patterning of physical activity behaviors, and in turn, obesity levels. | Results were divided into walkability/bike ability, green areas, and recreational facilities (playgrounds and sports). For walkability/bike ability, there were mixed results. In general, paths and facilities for walking and cycling tended to be better in areas with higher SES, while there were denser settlements and more destinations in areas with lower SES. Access to and distribution of green areas was highly variable without a clear trend. The same goes for playgrounds and sports facilities. | All |
| 67 | Jayasinghe, S., Flies, E.J., Soward, R., et al. (2021) *A Spatial Analysis of Access to Physical Activity Infrastructure and Healthy Food in Regional Tasmania.* | Australia | Spatial analysis, using QGIS data and on-ground and remote-sensing approaches to identify relevant physical activity infrastructure (PAI). | Analysis at area level, including three local goverment areas in Tasmania. | This research evaluated access (i.e., coverage, variety, density, and proximity) to physical activity resources and food outlets in relation to socioeconomic status (SES) in three NW Tasmanian communities. | In all three study areas, the PAI and food outlets were largely concentrated in the main urban areas with most recreational tracks and natural amenities located along the coastline or river areas. There was marked variation in accessibility to infrastructure across different areas of disadvantage within and between the sites with pockets of high socio-economic disadvantage in all three sites having some degree of poor accessibility to facilities. For a considerable proportion of the population, free-to-access natural amenities/green spaces and recreational tracks (73 and 57%, respectively) were beyond 800 m from their households. | Neighborhood parks and open spaces and Sports facilities |
| 84 | Karusisi, N., Thomas, F., Méline, J., et al. (2013). *Spatial accessibility to specific sport facilities and corresponding sport practice: the RECORD Study.* | France | Cohort study, using survey data (n=7.290), physical examination and GIS data on facility location and residential address. | Adults above 30 years | The aim of this study was to investigate the associations between the spatial accessibility to specific types of sports facilities and the practice of the corresponding sports (team sports, racket sports, swimming, and fitness) after carefully controlling for various individual socio-demographic characteristics and neighborhood socioeconomic variables. | High individual education and high household income were associated with the practice of racket sports, swimming or related activities, and fitness over the previous 7 days. The spatial accessibility to swimming pools was associated with swimming and related sports, even after adjustment for individual/contextual factors. The spatial accessibility to facilities was not related to the practice of other sports. High neighborhood income was associated with the practice of a racket sport and fitness. | Sports facilities |
| 74 | Kokolakakis, T., Lera-López, F., & Castellanos, P. (2014). *Regional differences in sports participation: The case of local authorities in England* | England | Cross sectional study, using national survey data (n=166.000) | Adults above 16 years | This paper investigates the determinants of sports participation at regional level in England. The study analyzes the differences in the regional characteristics among 325 English Local Authorities (LAs). | The analysis showed that differences in education, income, ethnicity, and population explained some of the variation in PA participation. The proportion of volunteers was also a significant factor, while access to sports facilities or municipal financial support for the area did not help to explain the differences. It is pointed out that facilities are not insignificant, but that the distribution between the municipalities is not great due to political and capitalist mechanisms. | Sports facilities |
| 80 | Lamb, K.E., Ogilvie, D., Ferguson, N.S., et al. (2012). *Sociospatial distribution of access to facilities for moderate and vigorous intensity physical activity in Scotland by different modes of transport.* | Scotland | GIS analysis on sport facilities in different SES-areas. | Analysis on area level | This study examines the socio spatial distribution of access to facilities for moderate or vigorous intensity physical activity in Scotland and whether such access differs by the mode of transport available and by Urban Rural Classification | Prior to adjustment for Urban Rural Classification and local authority, the median number of accessible facilities for moderate or vigorous intensity activity increased with increasing deprivation from the most affluent or second most affluent quintile to the most deprived for all modes of transport. However, after adjustment, the modelling results suggest that those in more affluent areas have significantly higher access to moderate and vigorous intensity facilities by car than those living in more deprived area. | Sports facilities |
| 92 | Langøien, L.J., Terragni, L., Rugseth, G., et al. (2017). *Systematic mapping review of the factors influencing physical activity and sedentary behaviour in ethnic minority groups in Europe: A DEDIPAC study.* | Norway, Sweden, the Netherlands, Germany, England, Denmark, France, Greece, and Scotland. | A systematic mapping review, including 41 quantitative- and 22 qualitative articles | Minority groups living in Europe | This study is mapping the factors influencing physical activity and sedentary behavior among ethnic minority groups living in Europe. | Available sports facilities, lack of appropriate activities and lack of culturally sensitive facilities were the most prevalent factors regarding the importance of the environment for physical activity for minority groups in European countries. | Sports facilities |
| 85 | Lee, R.E., Cubbin, C., & Winkleby, M. (2007). *Contribution of neighbourhood socioeconomic status and physical activity resources to physical activity among women.* | USA | Based on the Stanford Heart Disease Prevention Program from 1979-1990, using surveys, laboratory measures and residential addresses. This study uses data from survey 1 in 1979, survey 4 in 1985 and survey 5 in 1990. (n=2672) | Women between 25 and 75 years | This study aimed to investigate whether access to physical activity resources mediated the relationship between neighborhood socioeconomic status and physical activity among women. | After accounting for individual-level socioeconomic status, women who lived in lower-socioeconomic status neighborhoods reported greater energy expenditure, but undertook less moderate physical activity, than women in moderate-socioeconomic status neighborhoods. In contrast, women living in higher socioeconomic status neighborhoods reported more vigorous physical activity than women in moderate socioeconomic status neighborhoods. | Sports facilities |
| 86 | Liu, Y., Taylor, P., & Shibli, S. (2009). *Sport equity: benchmarking the performance of english public sport facilities.* | England | Repeated user surveys of sports facilities (n=408) in 1997, 2001, 2006 and 2007. | Facility users with a focus on five vulnerable groups: Young, elderly, disabled people with low socio-economic status and minority groups. | This paper aims to investigate how public sports facilities were used by five disadvantaged groups over the past ten years. | Older citizens (60+) and those with low SES are generally underrepresented in the use of sports facilities. Finally, facility type, location, size, and management type were found to be major sources of performance differences for certain indicators | Sports facilities |
| 59 | Martin, A., Morciano, M., & Suhrcke, M. (2021). *Determinants of bicycle commuting and the effect of bicycle infrastructure investment in London: Evidence from UK census microdata.* | England | Cross sectional survey data from the Office for National Statistics in UK in 2001 (n=151,245) and 2011 (n=182,495). | Employed people aged 16-74 years | Research question 1: To what extent can borough-level differences in the likelihood of cycling, and time-trends in the likelihood of cycling, be explained by individual-level SES and demographic characteristics, or by local geographic features? And 2) how can it be explained by differences in cycle infrastructure expenditure, after controlling for demographic and SES-related changes in the population structure? | The study observed differences and time trends in cycling prevalence were partially explained by area-level differences in topography, greenspace, footpaths, and crime levels and by differences and changes in population structures. The study further showed that expenditure on cycling infrastructure was associated with increased cycling at a marginal rate of £4915 per additional commuter cyclist, with some variation between groups: ethnic minorities were more responsive, and females, older people and those with lower socioeconomic status appeared less responsive. | Cyclist infrastructure |
| 37 | Mayne, D.J., Morgan, G.G., Jalaludin, B.B., et al. (2017). *The contribution of area-level walkability to geographic variation in physical activity: A spatial analysis of 95,837 participants from the 45 and Up Study living in Sydney, Australia.* | Australia | Cohort study using survey data from 2006 and 2010 (n=95837). | Adults above 45 years | This study examines the relationships between the walkability of an area and the respondents' physical activity. | Walkability defined as more densely built-up areas, with more destinations and a branched road network was correlated with walking and meeting the recommendations for physical activity. Individual factors such as education, income and age also influence physical activity, but the importance of the environment was similar across SES. | Walkability |
| 60 | Mears, M., Brindley, P., Barrows, P., et al. (2021). *Mapping urban greenspace use from mobile phone GPS data.* | England | GPS data collected through a mobile phone app and survey data on users of green spaces. | Adults between 18 and 71 years | This study analyzes the characteristics of trips to greenspaces and whether trip-level characteristics are associated with selected demographic characteristics (age, gender, ethnicity, and socioeconomic deprivation). | The study finds that local users of the app spend an average of an hour per week visiting greenspaces, including around seven trips per week. Trip characteristics vary with user demographics: ethnic minority users and users from more socioeconomically deprived areas tend to make shorter trips than White users and those from less deprived areas, while users aged 34 years and over make longer trips than younger users. Women, on average, make more frequent trips than men, as do those who spent more time outside as a child. | Neighborhood parks and open spaces |
| 81 | Panter, J., Jones, A., & Hillsdon, M. (2008). *Equity of access to physical activity facilities in an English city.* | England | A cross sectional geographical study using survey data (n=401) and GIS data about distance to facilities. | Adults above 16 years | This study seeks to examine associations between household income, access to sports facilities and gyms, and physical activity in an English city | The study finds that the poorer the local area you live in, the longer it is to sports facilities. It is also this group that has the lowest activity level, which may be due to poorer access to facilities. | Sports facilities |
| 77 | Pascual, C., Regidor, E., Arco, D.A., et al. (2013). *Sports facilities in Madrid explain the relationship between neighbourhood economic context and physical inactivity in older people, but not in younger adults: A case study.* | Spain | Cross sectional study, using survey data (n=6607) | Adults between 16 and 74 years | To evaluate whether the availability of sports facilities help explain the differences in physical inactivity according to the economic context of the neighborhood. | The poorest neighborhoods show the highest prevalence of physical inactivity. The availability of sports facilities explains an important part of this excess prevalence in participants aged 50–74 years, but not in younger individuals | Sports facilities |
| 14 | Pascual, C., Regidor, E., Astasio, P., et al. (2007). *The association of current and sustained area-based adverse socioeconomic environment with physical inactivity.* | Spain | Cross sectional study, using national health survey data from 2001 (n=19.324) and area data about economy (GDPpc) and the number of sport facilities (National Census og Sports Installations 1998). | Adults between 16 and 74 years | This study seeks to evaluates the association between socioeconomic environment in the province of residence and physical inactivity, using measures of current and sustained area-based adverse socioeconomic environment. | Analyzes showed that lower economic prosperity in the provinces is correlated with higher physical inactivity. At the same time, there was a high correlation between the economic prosperity of the provinces and the number of sports facilities. No direct interaction analyzes were performed, but the study suggests that the number of sports facilities is a good indicator of a province's economic prosperity in Spain. | Sports facilities |
| 83 | Pascual, C., Regidor, E., Martínez, D., et al. (2009). *Socioeconomic environment, availability of sports facilities, and jogging, swimming and gym use.* | Spain | Study using survey data (n=25.982) and information of sports facilities in each province, from the National Census of Sports Installations, carried out in 1999. | Adults between 25 and 74 years | The aim of the study was to evaluate the association of the availability of sports facilities and socioeconomic environment with jogging, swimming and gym uses in Spain. | The study finds that there are more swimmers and gym users in provinces with a better economy, while people in poorer provinces do more jogging. But there is no correlation with the number of facilities and the number of practitioners. | Sports facilities |
| 38 | Prince, S.A., Reed, J.L., Martinello, N., et al. (2016). *Why are adult women physically active? A systematic review of prospective cohort studies to identify intrapersonal, social environmental and physical environmental determinants.* | United Kingdom, USA, Australia, Sweden, Canada, Spain, Belgium, Netherlands, Taiwan, Germany, Finland, Denmark, Austria, and France | Systematic review of prospective cohort studies, including 97 articles. | Women between 18 and 65 years | This study aims to systematically review available evidence from prospective cohort studies to identify intrapersonal, social environmental and physical environmental determinants of moderate-to-vigorous intensity physical activity (MVPA) among working-age women. | Most studied determinants were intrapersonal in nature with very few examining environmental determinants. Overall positive and consistent influencers of MVPA among working-age women include higher self-efficacy, higher self-rated health, higher QOL and greater intentions and perceived behavioral control to be physically active. There was no association with education, marital status, income, stress, social norms and PA facilities (number and proximity). | Walkability, sport facilities |
| 72 | Reimers, A.K., Wagner, M., Alvanides, S., et al. (2014). *Proximity to Sports Facilities and Sports Participation for Adolescents in Germany.* | Germany | Cross sectional study using survey data (N=1768) and GIS data on sports facilities. | Children and young people between 4 and 17 years | This study seeks to assess the relationship between proximity to specific sports facilities and participation in the corresponding sports activities for adolescents in Germany. | The study showed that girls residing longer distances from the nearest gym were less likely to engage in indoor sports activities; a significant interaction between distances to gyms and level of urbanization was identified. Decomposition of the interaction term showed that for adolescent girls living in rural areas participation in indoor sports activities was positively associated with gym proximity. Proximity to tennis courts and indoor pools was not associated with participation in tennis or water sports, respectively. | Sports facilities |
| 82 | Riva, M., Gauvin, L., & Richard, L. (2007). *Use of local area facilities for involvement in physical activity in Canada: insights for developing environmental and policy interventions.* | Canada | Cross sectional study, using telephone surveys (n=1006) | Adults between 25 and 55 years | The purpose of this study was to examine the individual (sex, age, education, and extent of involvement in vigorous physical activity) and local area characteristics (socioeconomic status, locations, and number of physical activity organizations per 1000 residents) associated with the use of local facilities for involvement in physical activity. | The study shows that women use the local facilities more often if they live in smaller towns or more affluent areas or are in the young or older group; or are most active. This variation cannot be found for men's use of facilities, which indicates that local conditions may play a greater role for women than men in terms of facility use. | Sports facilities |
| 90 | Rovniak, L. S., Sallis, J. F., Saelens, B. E., et al. (2010). *Adults' Physical Activity Patterns Across Life Domains: Cluster Analysis With Replication.* | USA | This study is described as an observational epidemiologic study designed to increase understanding of factors that explain variation in population-level physical activity and is using survey data (n=1689) and accelerometer measures. | Adults between 20 and 65 years | This study aims to explore: (1) If adults form identifiable physical activity clusters across leisure, occupation, transport, and home domains; and (2) Objectively measured physical activity, psychosocial, and built environment differences between members of different physical activity clusters. | The study categorizes the participants into three clusters based on questionnaire data: Low activity; Active leisure; and Active job. The low activity group is generally a little active in all four life domains (transport, work, free time, and home), and the active leisure group is significantly more active during their leisure time, while the active job group is more physically active on the job. Participants categorized as actively employed were more often male, had slightly lower incomes and lived in areas with lower average incomes. The active leisure group had the highest number of minutes of objective measure physical activity and lower BMI. At the same time, they had significantly greater social support from friends and family and indicated fewer barriers and more benefits for physical activity. Regarding the surroundings the active leisure group indicated slightly more sports facilities in the immediate area. | Sports facilities |
| 25 | Rydenstam, T., Fell, T., Buli, B.G., et al. (2020). *Using citizen science to understand the prerequisites for physical activity among adolescents in low socioeconomic status neighborhoods - the NESLA study.* | Sweden | Audio narratives and pictures, GPS data and surveys (n=32) | Teenagers between 16 and 19 years from two low SES areas in Sweden. | This study investigates physical activity in relation to  the built and social environments in low SES neighborhoods in a Swedish setting. | Among citizens from low SES neighborhoods, the most frequently reported facilitators were ‘parks, playgrounds and outdoor gym’ as well as ‘amenities’ and ‘sport facilities’, whereas lack of or shortcomings regarding ‘bike ability and walkability’, ‘personal safety’ and ‘lighting’ were the most frequently reported barriers. | Walkability and Cyclist Infrastructure |
| 31 | Salvo, G., Lashewicz, B.M., Doyle-Baker, P.K., et al. (2018). *Neighbourhood built environment influences on physical activity among adults: A systematized review of qualitative evidence.* | Australia, Canada, USA, New Zealand, Sweden, Iceland, United Kingdom, Belgium, Brazil, and Ireland | Systematic review including 36 peer-reviewed qualitative studies | Adults | This study is exploring how the built environment influences physical activity in adults. | The 36 studies have different populations in terms of gender, age, and ethnicity. Across these, the authors find that safety from crime and in traffic is most often mentioned. In addition, suitable destinations both in terms of active transport and in terms of sports participation are also widely discussed. Finally, aesthetic, and functional factors are also mentioned in approx. half of the studies. This includes, for example, green areas and the maintenance of paths and pavements. The authors also mention that the environment can have greater or perhaps directly opposite significance for different groups. Among other things, much emphasis on the elderly's opportunities for a safe and secure neighborhood and destinations close by, and for ethnic minorities' opportunity to be physically active in appropriate cultural contexts. | Walkability, Cyclist infrastructure and Neighborhood parks and open spaces |
| 64 | Schneider, R.A., Smith, A.M., Bullas, H., et al. (2020). *Multiple deprivation and geographic distance to community physical activity events — achieving equitable access to parkrun in England.* | England | The study is a cross sectional ecological analysis, using geolocation data and index of Multiple Deprivation. | Analysis on area level and with area specific data | The objective of this study is to evaluate geographic access to free weekly outdoor physical activity events (‘parkrun’) in England, with a particular focus on deprived communities, and to identify optimal locations for future events to further maximize access. | 69% of the population lived within 5 km of a parkrun event. Creating 200 new events in the recommended (optimal) green spaces would further improve access, increasing this to 82%. Contrary to the researcher’s expectation, they found that geographic access is slightly better for those living in more deprived communities. | Neighborhood parks and open spaces |
| 44 | Seguin-Fowler, R.A., LaCroix, A.Z., LaMonte, M.J., et al. (2022). *Association of neighborhood Walk Score with accelerometer-measured physical activity varies by neighborhood socioeconomic status in older women.* | USA | CO-hort study including survey data (n=5,650) | Adult women (average age 79,5 years.) | This study examines associations between neighborhood Walk Score and objective physical activity behavior in older adult women. They also examined the relationship between Walk Score and physical activity by neighborhood socioeconomic status. | People living in areas with a high smart street walk score take more steps per day. The association between walk score and physical activity varied by neighborhood socioeconomic status. Among those living in low socioeconomic status neighborhoods, there was no association between walk score and physical activity. In contrast, among high neighborhood socioeconomic status, higher walk score (i.e., less car dependent) was significantly positively related to increased steps per day. In high socioeconomic status neighborhoods, participants in walker’s paradise locations had 1175.9 extra steps per day compared to participants in car-dependent locations. | Walkability |
| 55 | Smith, M., Hosking, J., Woodward, A., et al. (2017). *Systematic literature review of built environment effects on physical activity and active transport - an update and new findings on health equity.* | Australia, USA, Belgium, United Kingdom, Scotland, and New Zealand. | Systematic review of quantitative empirical interventions studies (n=28) | Children and adults | The aims of this systematic review were to identify which environmental interventions increase physical activity in residents at the local level, and to build on the evidence base by considering intervention cost, and the differential effects of interventions by ethnicity and socioeconomic status. | Despite the relatively few and diverse studies, most studies find an effect of the interventions (visits, use or physical activity). The most promising interventions are walking and cycling facilities, adding fitness equipment, paths, or lighting, and increasing sports facilities. Out of the 28 studies, 4 of them examine differences between population groups. One study finds increased use of renovated parks for white Americans, but not for colored Americans. A study finds a difference in the use of new cycling infrastructure in relation to the participants' income, but not a difference in cycling or walking behavior. In the last two studies - concerning the use of a new cycle path and sports participation because of more sports facilities - no difference in the effect was found. | Walkability and Cyclist infrastructure |
| 68 | Spencer, L. H., Lynch, M., Lawrence, C. L., et al. (2020). *A scoping review of how income affects accessing local green space to engage in outdoor physical activity to improve well-being: Implications for post-COVID-19.* | USA, Canada, Corea, South Africa, China, and United Kingdom | Scoping review (n=15) | N/A | The purpose of this review was to understand if income had an influence on people accessing green spaces within local environments to undertake physical activity (PA). | The findings from strongly indicate there is an effect of income on using green spaces for PA, but the relationship is erratic and there are many characteristics of the environment, perceived or otherwise, that influence whether an individual engages in PA in local neighborhood environments or not. | Neighborhood parks and open spaces |
| 34 | Sun, P., & Lu, W. (2022). *Environmental inequity in hilly neighborhood using multi-source data from a health promotion view.* | China | Multi-source analysis using survey data, street design elements and topographic measurements. | Residents from different SES areas | This paper investigates environmental inequalities in a hilly urban environment in the context of the booming real estate market in China, comprised of health promotion-related elements, namely, built environment, physical activity facilities, street infrastructure, green spaces, and environmental perceptions. | Housing price was positively correlated with facilities, green space, and positive environmental perceptions. These coefficients indicated the intensity of the association was different over different travel distances. Additionally, living density was positively related to facility availability and quality. The correlation between residential density and physical activity facilities was stronger than the housing price. This result suggested that less well-maintained communities (i.e., lower-level communities) tended to have a smaller supply of facilities. And residents living in the highest socioeconomic community level had access to more activity facilities within the same distance, with better quality facilities and a greater variety of street furniture. In terms of aesthetics needs, high-grade communities could get a better view of the street greenery and have a better feeling of aesthetics and safety in the street environment | Walkability, Neighborhood parks and open spaces and Sports facilities |
| 52 | Todd, M., Adams, M.A., Kurka, J., et al. (2016). *GIS-measured walkability, transit, and recreation environments in relation to older Adults' physical activity: A latent profile analysis.* | USA | This study is using survey data (n=714), an observational study and GIS data. | Elderly between 66-97 years | The study seeks to derive empirically defined latent profiles of seven GIS-measured BE features using pooled data from a sample of older adults residing in two U.S. metropolitan regions, and secondly examine how neighborhood profiles were related to objectively measured MVPA (measuring total PA volume and intensity) and self-reported PA (measuring specific types of PA engaged in, e.g., walking for errands), objectively measured sedentary time, and BMI, which is related to both MVPA and overall health in older adults | Three area profiles – low, medium, and high walkability/transit/recreation. There are more active older people in areas with high walkability/transit/recreation – but only 7% of the participants live here. Minor (not significant) differences between medium walkability/transit/recreation in the built environment and low walkability/transit/recreation in the built environment. There is thus a correlation between how active the elderly are and the ease of walking/transit/recreation in the built environment. | Walkability |
| 15 | Wang, S., Liu, Y., Lam, J., et al. (2021). *The effects of the built environment on the general health, physical activity, and obesity of adults in Queensland, Australia.* | Australia | Empirical study, based on survey data (n=1,788) and GIS data in relation to areas | Adults between 18 and 65 years | This study aims to examine the associations between individual characteristics, the built environment, physical activity, general health, and body mass index to reveal the pathways through which the built environment is associated with the prevalence of  obesity. | Neighborhoods with less car independence, low road coverage and housing density, fewer commuters by public transport, and diverse housing are associated with a higher level of physical activity; and this is also the case for neighborhoods with less land-use diversity and longer distance to the city of Brisbane. They further find that neighborhoods with more green space and a higher level of socioeconomic status tend to be associated with a higher level of physical activity. | Walkability and Neighborhood parks and open spaces |
| 91 | Werneck, A.O., Oyeyemi, A.L., Araújo, R.H.O., et al. (2022). *Association of public physical activity facilities and participation in community programs with leisure-time physical activity: does the association differ according to educational level and income?* | Brazil | Cross sectional study using survey data from the Brazilian National Health Survey (n=88,531). | Adults above 18 years | Our aim was to analyze the association of the presence of public physical activity (PA) facilities and participation in public PA programs with leisure-time PA, with an emphasis on the moderating role of educational level and income. | The presence of public PA facilities near the household and the participation in public PA programs were  associated with higher leisure-time PA among all quintiles of income and educational level. However, multiplicative  interactions revealed that participating in PA programs and the presence of public PA facilities near the household were more associated with higher odds of being active in the leisure-time among the lowest quintile of educational level. | Sport facilities |
| 45 | Zang, P., Xian, F., Qiu, H., et al. (2022). *Differences in the Correlation between the Built Environment and Walking, Moderate, and Vigorous Physical Activity among the Elderly in Low- and High-Income Areas.* | China | Cross sectional study using survey data (n=597) | Elderly over 65 years from respectively low SES and high SES areas | In this study, two types of regions were divided according to different socioeconomic levels of Guangzhou (China). Then, the differences in walking, moderate, and vigorous physical activity among the elderly were compared in combination with the population density at the high, middle, and low levels. | It was clear that walking, moderate, and vigorous physical activity times were generally higher in high socioeconomic areas than in low socioeconomic areas, and, conversely, the sedentary time was generally higher for older people in low socioeconomic areas than in high socioeconomic areas. The PA of people living in low-SES areas was more dependent on the built environment, whereas the correlation may be limited in high SES areas. Moreover, in low SES areas, walking was negatively correlated with street connectivity; moderate PA was positively correlated with street connectivity and the shortest distance to the subway station, but negatively correlated with the density of entertainment points of interest (POIs). | Walkability |
| 62 | Zhang, R., Zhang, C.Q., Lai, P.C., et al. (2021). *Park environment and moderate‑to‑vigorous physical activity in parks among adolescents in a high‑density city: the moderating role of neighbourhood income.* | China | Cross sectional study, making observations in 32 urban parks (SOPARC) N=2397 | Adolescents | The current study aimed to examine the associations between park environmental characteristics and moderate-to-vigorous physical activity (MVPA) in parks among adolescents in Hong Kong and the moderating effect of neighborhood income on these associations. | There was a significant positive association between the quality of amenities and park based MVPA (metabolic equivalents per observation) in adolescents. However, the associations between the diversity of active facilities, greenness and adolescents’ park based MVPA were not significant. Neighborhood income moderated the association between adolescents’ park based MVPA and park safety, where the relationship between park safety and park based MVPA was significantly positive in low-income neighborhoods but not significant in high-income neighborhoods. An income-by-environment interaction was also observed concerning park aesthetics, with a negative relationship between park aesthetics and park-based MVPA in high-income neighborhoods but not in low-income neighborhoods. | Neighborhood parks and open spaces |
